# Co-assembly of precision polyurethane ionomers reveals role of and interplay between individual components†

**DOI:** 10.1039/d1py00079a

**Published:** 2021-04-22

**Authors:** Elizabeth M. Timmers, P. Michel Fransen, Álvaro González García, Sandra M. C. Schoenmakers, Jose Rodrigo Magana, Joris W. Peeters, Ronald Tennebroek, Ilse van Casteren, Remco Tuinier, Henk M. Janssen, Ilja K. Voets

**Affiliations:** Laboratory of Self-Organizing Soft Matter, Department of Chemical Engineering and Chemistry, Eindhoven University of Technology P.O. Box 513 5600 MB Eindhoven The Netherlands i.voets@tue.nl; Laboratory of Macro-Organic Chemistry, Department of Chemical Engineering and Chemistry, Eindhoven University of Technology P.O. Box 513 5600 MB Eindhoven The Netherlands; Institute for Complex Molecular Systems, Eindhoven University of Technology P.O. Box 513 5600 MB Eindhoven The Netherlands; SyMO-Chem B.V. Den Dolech 2 5612 AZ Eindhoven The Netherlands; Laboratory of Physical Chemistry, Department of Chemical Engineering and Chemistry, Eindhoven University of Technology P.O. Box 513 5600 MB Eindhoven The Netherlands; Van ‘t Hoff Laboratory for Physical and Colloid Chemistry, Department of Chemistry and Debye Institute for Nanomaterials Science, Utrecht University Padualaan 8 3584 CH Utrecht The Netherlands; DSM Resins and Functional Materials Sluisweg 12 5145 PE Waalwijk The Netherlands

## Abstract

Industrial and household products, such as paints, inks and cosmetics usually consist of mixtures of macromolecules that are disperse in composition, in size and in monomer sequence. Identifying structure–function relationships for these systems is complicated, as particular macromolecular components cannot be investigated individually. For this study, we have addressed this issue, and have synthesized a series of five sequence-defined polyurethanes (PUs): one neutral-hydrophobic, one single-charged hydrophilic, one single-charged hydrophobic and two double-charged amphiphilic PUs (one symmetric and one asymmetric). These novel precision PUs – that were prepared by using stepwise coupling-deprotection synthetic protocols – have a defined composition, size and monomer sequence, where the chosen sequences were inspired by those that are abundantly formed in the production of industrial waterborne PU dispersions. By performing dynamic light scattering experiments (DLS), self-consistent field (SCF) computations and cryogenic transmission electron microscopy (cryo-TEM), we have elucidated the behavior in aqueous solution of the individual precision PUs, as well as of binary and ternary mixtures of the PU sequences. The double-charged PU sequences (‘hosts’) were sufficiently amphiphilic to yield single-component micellar solutions, whereas the two more hydrophobic sequences did not micellize on their own, and gave precipitates or ill-defined larger aggregates. Both the neutral-hydrophobic PU and the hydrophilic single-charged PU were successfully incorporated in the host micelles as guests, respectively increasing and reducing the micelle radius upon incorporation. SCF computations indicated that double-charged symmetric PUs stretch whilst double-charged asymmetric PUs are expelled from the core to accommodate hydrophobic PU guests within the micelles. For the ternary mixture of the double-charged symmetric and asymmetric hosts and the neutral-hydrophobic guest we have found an improved colloidal stability, as compared to those for binary mixtures of either host and hydrophobic guest. In another ternary mixture of precision PUs, with all three components not capable of forming micelles on their own, we see that the ensemble of molecules produces stable micellar solutions. Taken together, we find that the interplay between PU-molecules in aqueous dispersions promotes the formation of stable micellar hydrocolloids.

## Abstract 2

In a novel synthetic approach, we have prepared sequence-defined waterborne polyurethanes and have studied their (co)-assembly to hydrocolloids; we find that especially the interplay between the sequences promotes the formation of stable micelles.

## Graphical abstract

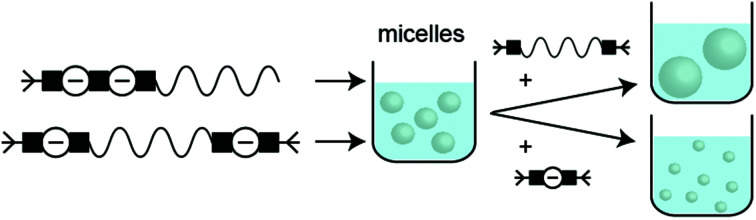

## Introduction

The function of biopolymers such as peptides, proteins, and nucleic acids often relies on the hierarchical assembly of multiple compounds into supramolecular architectures.^1^ Examples of self-assembly, *e.g.* enzymes that are activated by dimerization, or co-assembly, *e.g.* proteins that dock onto DNA to regulate transcription, are ubiquitous. Indeed, intricate assembly pathways in natural systems lead to well-defined complexes capable of performing virtually all functions essential for life.^1,2^ Similarly, when closely examining man-made industrial and household products and materials, such as foods, drugs, paints and coatings, one may also find that hierarchical assembly processes of multiple components within these systems play a pivotal role in the function and properties of the final products.

Most studies on the association in solution of synthetic (macro)molecules focus on well-defined (model) systems, usually involving compounds that are examined under controlled conditions.^3,4^ Particularly well-studied is the self-assembly of both low and high molar mass amphiphiles into core–shell micelles.^3,4^ Far less is known about the co-assembly of mixtures of synthetic (macro)molecular components, as for several reasons such systems have been studied less frequently. Firstly, synthetic polymers are not as well-defined molecularly as, for example, low molecular weight surfactant compounds or biopolymers: synthetic polymers are generally disperse in composition, in size and in monomer sequence. These aspects have important consequences for their assembly^5^ and co-assembly properties. Secondly, the in-depth characterization in solution of mixtures of species of comparable, nanometric size is demanding, as contributions of the individual species with small differences in dimensions and properties are challenging, if not impossible, to resolve.

An appealing strategy to advance the understanding of the behavior of multicomponent mixtures is to prepare precision polymers with as little dispersity in composition, sequence, and size as possible and use these to prepare well-defined mixtures for in-depth characterization. In the past decade, this approach has gained increasing attention, for example in a study on the impact of size dispersity on the assembly of block copolymers and oligomers in bulk and in solution.^5^ Two main strategies to prepare (macro)molecules low in dispersity, size and sequence are (i) stepwise synthetic approaches and (ii) controlled radical polymerization (CRP) methods.^2^ These procedures yield so-called ‘sequence-defined’ and ‘sequence-controlled’ polymers, respectively. Stepwise synthetic routes are akin to solid-phase peptide synthetic approaches that were introduced to prepare perfectly defined oligopeptides from canonical amino acids,^6^ and such routes can be used to produce polymers and oligomers from other synthetic monomer origin as well.^7^ Stepwise methods yield truly discrete compounds termed ‘sequence-defined’ polymers, such as sequence-defined polypeptoids,^8^ polyamides^2^ and oligocarbamates.^7^ Unfortunately, only lower molecular weight materials, essentially oligomers, are accessible, which is not surprising in view of the upper limit of ∼50 monomers in solid-phase peptide synthesis.^7^ By contrast, CRP allows the synthesis of high molecular weight polymers. However, CRP always yields slightly disperse polymer products which are therefore termed ‘sequence-controlled’. Overall, this research field has advanced tremendously, and truly sequence-defined polymers of 15 and 17 kDa have now been successfully produced,^9,10^ opening up opportunities for applications that require higher molecular weight precision materials.

Polyurethanes (PUs) constitute an important class of polymer materials which find use in a broad spectrum of applications ranging from coatings and paints to high-performance elastomers to tough, rigid plastics.^11,12^ Traditionally, PU dispersions contained significant amounts of volatile organic solvents to facilitate processing, but in recent years waterborne polyurethanes (WPUs) have attracted great interest as useful alternatives with reduced adverse health and environmental impact.^11,12^ WPUs are typically dispersions of polyurethane ionomers (PUIs) that are produced in a two-step synthesis. In the first step, isocyanate-terminated prepolymers are prepared by reacting polyols (typically diols, *e.g.* poly-THF or poly-tetrahydrofuran diols) and ionizable monomers (typically dimethylpropionic acid, *i.e.* DMPA, a diol) with di- or tri-isocyanates (typically di-isocyanates, *e.g.* IPDI or isophorone diisocyanate).^12^ The result is a mixture of isocyanate terminated intermediates, of which the exact composition is determined by statistics. In the second step, the prepolymers are dispersed in water at a high pH (accordingly, the DMPA-units become anionic), and are chain-extended using a polyamine (typically a di-amine). Prior to chain-extension, the prepolymer solution is a multi-component mixture of (macro)molecules with different amounts of ionizable groups, molecular weights, and hydrophobicities. The components may or may not be surface active, and may co-assemble or instead segregate into different types of hydrocolloids. Desired solubilization into stable hydrocolloids (before and after chain-extension), as opposed to unwanted precipitation of components, is often accomplished by performing empirical (‘trial and error’) studies, and this approach has led to great progress and knowledge in PU formulation, application and product design.

Given their synthesis, industrial WPU dispersions comprise a mixture of macromolecules with a variety in composition, monomer sequence and length. In addition to amphiphilic compounds with a tendency to associate into micelles, WPU mixtures may contain fairly soluble, hydrophilic species and rather insoluble, hydrophobic compounds. In contrast to conventional amphiphiles and surfactants where one hydrophobic and one hydrophilic segment are simply joined together head-to-tail, PUI macromolecules have hydrophilic (DMPA) and hydrophobic units (isophorone diamine (IPDA), poly-THF) randomly distributed along the polymer chain. In prior work, sequence-controlled dendritic PUs have been obtained *via* a selective reactivity approach; the products were non-ionic and can therefore not be dispersed in water.^13^ In this work, we have set out to prepare sequence-defined PUIs of variable composition, size and monomer sequence to reflect the breadth of the various species present in industrial WPU dispersions. This should allow for in depth studies on these PUI macromolecules.

**Fig. 1:**
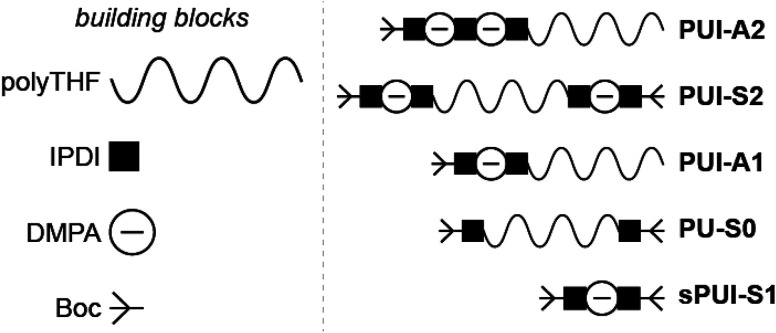
Schematic representation of the sequence-defined precision PU(I)s as used in this study, and their constituting building blocks: poly(tetrahydrofuran) (polyTHF) with an average molar mass of 2 kDa, isophorone di-isocyanate (IPDI), dimethylpropionic acid (DMPA), and the *tert*-butyloxycarbonyl protecting group (Boc). Polyurethane ionomers **PUI-A1** and **PUI-A2** are asymmetric with one (**PUI-A1**) or two (**PUI-A2**) chargeable DMPAs, respectively. Polyurethane ionomer **PUI-S2** is symmetric and bears one DMPA group on either poly-THF chain end. Polyurethane **PU-S0** is symmetric, does not contain DMPA and is therefore neutral and hydrophobic. The small polyurethane ionomer **sPUI-S1** bears one charge and does not contain a polyTHF chain.

Accordingly, and aiming to elucidate the functional role of the various polyurethane molecules in industrially relevant multicomponent WPU mixtures (prior to chain extension), we have created a small but representative series of five sequence-defined PUs (or precision PUs, see Fig. 1 and Scheme S1†). The novel precision PU(I)s have been synthesized through a stepwise coupling-deprotection protocol using poly-THF (2 kDa), IPDA and/or DMPA building blocks. Next, we have studied their association behavior in aqueous solution, either per PU sequence individually or in combination with one or two of the other PU sequences in mixtures of controlled composition. Previously, SCF calculations revealed that the specific surface active **PUI-A2** and **PUI-S2** sequences are relatively abundant in PU dispersions and presumably play a major role in particle formation and stabilization in WPUs.^14^ However, to date such materials had not been prepared and tested experimentally. Dynamic light scattering (DLS) experiments and self-consistent field (SCF) computations have been used to investigate individual PUs or multicomponent PU mixtures with exactly known composition. In addition, cryogenic transmission electron microscopy (cryo-TEM) has been used to visualize the hydrocolloids formed by the PUs. The employed combination of experimental and computational tools, previously used to study other surface active materials, allows to investigate dissolution, micellar dimensions, colloidal morphology, influences of used co-solvents, chain packing, and location of the different types of chains within mixed micelles.^15,16^ Our findings reveal whether individual PU sequences preferentially reside in the micellar interior (core) or exterior (corona). Furthermore, we find that micelles co-assembled from two or three PUs display an increased encapsulation efficiency of the non-soluble hydrophobic PU guest, as compared to hydrocolloids self-assembled from single PU components. Accordingly, we have seen that mixed micelles of PUs display improved colloidal stabilities.

## Methods

### Synthesis and molecular characterization of precision polyurethanes

Experimental details on the preparation of the PU materials used in this study have largely been described previously (**PU-S0**, **PUI-S2** and **PUI-A2**).^17^ The synthetic details of the asymmetric ionomer with one DMPA group (**PUI-A1**) and the small polyurethane ionomer with one DMPA group (**sPUI-S1**) are described in the ESI.† Analytical details on all five PUs, *i.e.***PU-S0**, **PUI-S2**, **PUI-A1**, **PUI-A2** and **sPUI-S1**, are compiled in the ESI.† These details include data on nuclear magnetic resonance (NMR) spectroscopy, attenuated total reflection Fourier-transform infrared (ATR-FT-IR) spectroscopy, size exclusion chromatography (SEC), high-performance liquid chromatography mass spectrometry (HPLC-MS) and matrix-assisted laser desorption ionization time-of-flight mass spectrometry (MALDI-TOF-MS). Finally, thermal data on the materials, as collected by differential scanning colorimetry (DSC), are provided in the ESI.†

### Light scattering experiments

#### Sample preparation

Dispersions of individual or mixed PU(I)s in water were prepared as follows. First, the PU(I)s were individually dissolved in tetrahydrofuran (THF) stock solutions at a PU(I) weight concentration of 0.5–5.0 mg mL^−1^. These solutions in THF were subsequently mixed at the desired PU(I) ratio for each sample and pipetted into 10 mL glass vials. The total THF volume utilized ranged between 0.2 and 2.0 mL. Hereafter, the THF solvent was removed by spontaneous evaporation at room temperature, followed by removal of residual THF by evaporation *in vacuo* at 40 °C to form thin PU(I) films at the bottom and walls of the glass vial. The PU(I) films were subsequently rehydrated by addition of a 0.1 M solution of triethylamine (TEA) in water (pH ∼ 11), after which the samples were briefly vortexed at room temperature to facilitate even dispersion. In all samples, the final concentration of the main (host) PU(I) component(s) was kept constant at approximately 2.5 mg mL^−1^. The concentration of the additional PU(I) component, to which we refer in the remainder of this manuscript as ‘guest’, was varied, and is reflected in the guest loading (*f*_guest_) that is defined as:1
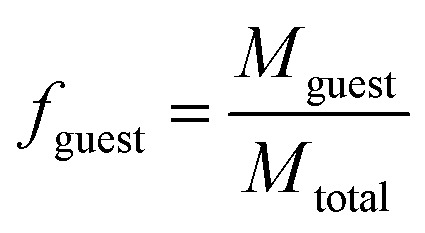
with *M*_guest_ and *M*_total_ being the mass of the guest PU(I) and the total PU(I) mass in the final samples (*i.e. M*_host(s)_ + *M*_guest_), respectively. After dissolution in the 0.1 M TEA aqueous solution, the samples were allowed to equilibrate for a few hours followed by filtration over a 0.45 μm filter (PTFE, Whatman) prior to the dynamic light scattering (DLS) experiments. All studied samples, prior to and after filtration, did not show precipitation, and were either clear or slightly hazy (unless specifically mentioned otherwise). The filtration was nevertheless performed to remove large particles, if any, not originating from any of the PU(I) components. According to the above, the loss of PU(I) material due to filtration was assumed negligible. For every encapsulation experiment, two series of sample solutions were prepared and analyzed by DLS.

### Dynamic light scattering (DLS)

DLS measurements were performed at 20 °C on a Malvern Zetasizer Micro V (μV), equipped with a 60 mW 830 nm laser. The second-order correlation function, *g*_2_(*t*) and total averaged scattered intensity, *I*_90_, were recorded at a fixed scattering angle *θ* = 90° for 60 seconds, in duplicate. As the samples are dilute and the PU(I) micelles small (*vide infra*), we assume little to no *q*- and concentration-dependence. Malvern Zetasizer 6.12 software based on the CONTIN algorithm was used to analyze the DLS data, obtaining the (collective) translational diffusion coefficient, *D*, and subsequently the hydrodynamic radius, *R*_h_, using the Stokes–Einstein equation2
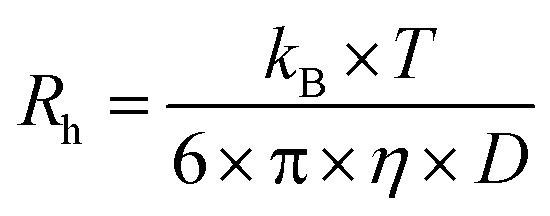
with the Boltzmann constant, *k*_B_, temperature, *T*, and the dynamic viscosity of the solvent, *η*.

### Cryogenic transmission electron microscopy (cryo-TEM)

Dispersions of individual or mixed PU(I)s in water were prepared as given above in the “Light scatttering experiments” section. Accordingly, all samples were aqueous 0.1 M TEA solutions with a concentration of the PUI host component(s) of approximately 2.5 mg mL^−1^. Vitrified films were prepared in a ‘Vitrobot’ instrument (FEI VitrobotTM Mark IV, FEI Company) at 22 °C and at a relative humidity of 100%. In the preparation chamber of the ‘Vitrobot’, 3 μL samples were applied on Quantifoil grids (R 2/2, Quantifoil Micro Tools GmbH), which were surface plasma treated just prior to use (Cressington 208 carbon coater operating at 5 mA for 40 s). Excess sample was removed by blotting using filter paper for 3 s with a blotting force of −1, and the thin film thus formed was plunged (acceleration about 3 g) into liquid ethane just above its freezing point. Vitrified films were transferred into the vacuum of a CryoTITAN microscope equipped with a field emission gun that was operated at 300 kV, a post-column Gatan energy filter, and a 2048 × 2048 Gatan CCD camera. Virtrified films were observed in the CryoTITAN microscope at temperatures below −170 °C. Micrographs were taken at low dose conditions at magnifications of 24000x (decofus = −10 μm) and 48000x (decofus = −5 μm). Dimensions of observed micelle particles were assessed by applying ImageJ software, an open source image processing program, by checking every particle twice, first along the longest axis of the particle and second perpendicular to this axis.

### Self-consistent mean field computations (SCF)

Following previously developed models for PU(I)s,^14^ the Scheutjens-Fleer self-consistent mean-field theory (SCF) was applied to predict equilibrium self-assembly properties of micelles. Recently, we have adopted the scheme in Li *et al.* to study the self-assembly of surfactants,^16^ and we follow the specifics therein indicated. In this work, we used a spherical lattice with concentration gradients in the radial direction only. Each lattice position corresponds to a spherical shell from the center of the lattice. The SFbox software, developed by prof. F.A.M. Leermakers of Wageningen University, the Netherlands, was used to conduct all SCF computations.^18,19^ The different components considered and the Flory—Huggins χ-parameters between them are presented in Table S6,† and both are based on previous investigations.^14,16^ Each lattice site is occupied by one segment, one ion or water. Due to the high pH conditions used in this study, we assumed that the carboxylic acid groups were deprotonated in all our computations. The lattice size was set to 0.36 nm and was also used as input to account for electrostatic interactions.^16^

The SCF follows a grand-canonical approach in which, provided a certain initial molecular configuration, the free energy of the lattice is minimized. The computed grand-potential *Ω* depends on the concentration of compounds. The aggregation number *g*_k_ is given by the excess number of molecules of compound *k* in the self-assembly. By combining small system thermodynamics with the SCF computations it is possible to identify the thermodynamically preferred self-organized morphology in terms of the amount of a certain compound in the lattice at which *Ω* = 0, with ∂*Ω*/∂*g*_k_ < 0. For every computation, the incompressibility constraint is enforced. At the most probable micelle condition, radial concentration profiles (*φ*) are collected. From these, the hydrodynamic micelle size can be calculated.^19^ The theoretical guest loading, *f*_guest,SCF_, was defined as3
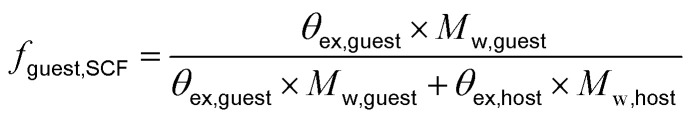
with the excess amounts of guest and host molecules, *θ*_ex,guest_ and *θ*_ex,host_, and the molecular weight of the guest and host, *M*_w,guest_ and *M*_w,host._

## Results and discussion

### Synthesis of sequence-defined polyurethanes and polyurethane ionomers

Industrially applied synthetic procedures to prepare WPUs produce a complex mixture of polyurethanes (PU) and polyurethane ionomers (PUI). The delicate balance between these PU substances, both in terms of their molecular structures and their abundance, controls the physicochemical properties of the resulting WPU dispersion. The understanding of the role of individual molecular components in the WPU materials, however, is scarce. Consequently, the design of new WPU formulations is highly challenging, and is often done by ‘trial-and-error’ experiments.

**Fig. 2:**
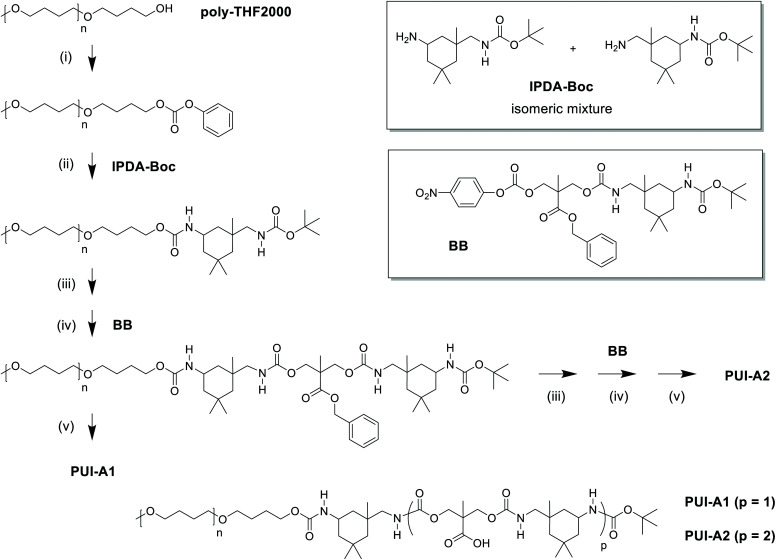
Synthetic approach to the preparation of sequence-defined PUIs, as illustrated by the route to **PUI-A1** and **PUI-A2**: (i) active carbonate formation by reaction with phenyl chloroformate; (ii) urethane formation by reaction with IPDA-Boc; (iii) amine formation by deprotection of Boc-group; (iv) urethane formation by chain extension with one BB unit; (v) carboxylic acid formation by deprotection of benzylester group. Similarly, and starting from telechelic poly-THF2000, **PU-S0** and **PUI-S2** have been prepared. IPDA-Boc was prepared in one step from IPDA; building block BB was prepared from DMPA and IPDA-Boc in three steps. Note that the IPDA building blocks exist in multiple regio- and stereoisomers; these are not shown for brevity.

In this work, we have synthesized a series of chemically distinct, sequence-defined PU(I)s (Fig. 1; Scheme S1†) to investigate the role of such molecules in the formation of stable hydrocolloids, more particularly micelles. We have used protective group chemistry applying an iterative coupling-deprotection strategy to produce the PU(I)s. The synthetic route to **PUI-A1** and **PUI-A2** is shown schematically in Fig. 2. In an analogous approach, and starting from bi-functional poly-THF, we have prepared **PUI-S0** and **PUI-S2**. The smaller **sPUI-S1** material was synthesized in five steps from dimethylpropionic acid (DMPA) and isophorone diamine (IPDA). As can be appreciated from the followed route, we have employed the reaction between amines and activated carbonates to create urethane linkages, and have avoided using the typical chemistry between alcohols and isocyanates to produce PU(I)s. Amines as well as activated carbonates are stable, so both these type of building blocks can easily be manipulated, purified and stored. In contrast, the relatively high reactivity of isocyanates towards water renders isocyanate intermediates effectively unstable, either during synthesis, work-up or storage, precluding their use in step-by-step preparations of precision materials.

**Fig. 3:**
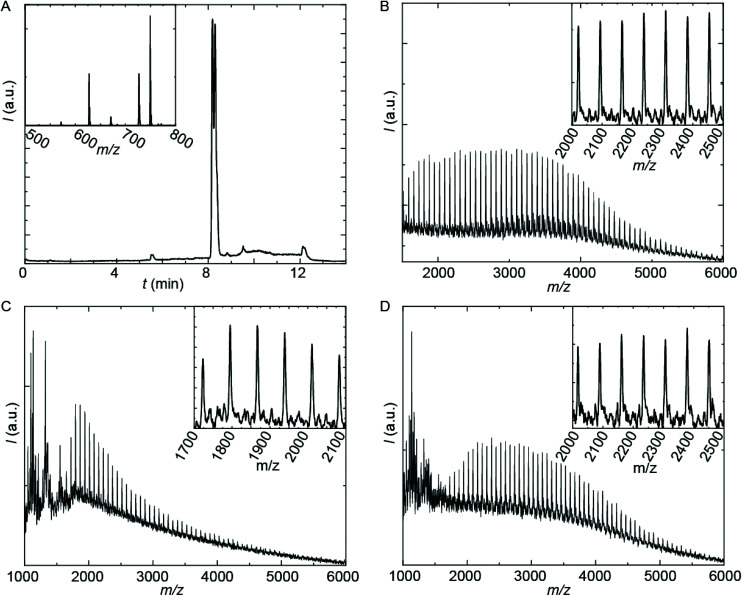
Mass spectrometry analysis of prepared PUIs. (A) HPLC-MS trace of **sPUI-S1**; the inset is the MS-spectrum of the dominant peaks at about *t* = 8.2 minutes showing [M + H^+^], [M + Na^+^] and [M-Boc + H^+^] adducts. (B-D) MALDI-TOF-MS spectra of **PUI-A1** (B), **PUI-S2** (C) and **PUI-A2** (D). Matrix: CHCA with added KAc. Mode: positive linear. Insets are zoom-ins. Enlarged pictures with details on the recorded masses in the arrays are collected in the ESI.

The employed organic synthetic approach made it possible to isolate PU(I)s with a strictly defined order of components, *i.e.* with a sequence-defined microstructure. In the prepared materials, the number of IPDA, DMPA and poly-THF groups is precisely controlled, as well as the positioning of these groups within the macromolecular structure. Note, however, that the produced PU(I)s are not chemically pure, where this is due to (i) the used poly-THF component, introducing a distribution and dispersity in molecular weight that is common for almost all synthetic polymers, and (ii) the employed IPDA component, introducing regio- and stereo-isomeric diversity. The latter is illustrated for IPDA-Boc in Fig. 2, but is not shown for the other PU(I)s for reasons of brevity. The five PU(I)s were molecularly characterized using NMR, ATR-FT-IR, SEC, HPLC-MS and MALDI-TOF-MS (see the ESI for full details†), and found data were in line with the assigned structures. Particularly, HPLC-MS analysis of **sPUI-S1** showed two dominant peaks for the isomers of this compound (Fig. 3A), while MALDI-TOF-MS analysis of **PUI-A1**, **PUI-A2** and **PUI-S2** showed single arrays of peaks corresponding to masses of individual oligomers for the respective PUIs (Fig. 3B–D), proving the integrity of the prepared materials. For all three polymers, the periodicities of the arrays were 72 Dalton, which is the molecular weight of a THF-monomer.

### Solution behavior of individual sequence-defined PU(I)s

**Fig. 4:**
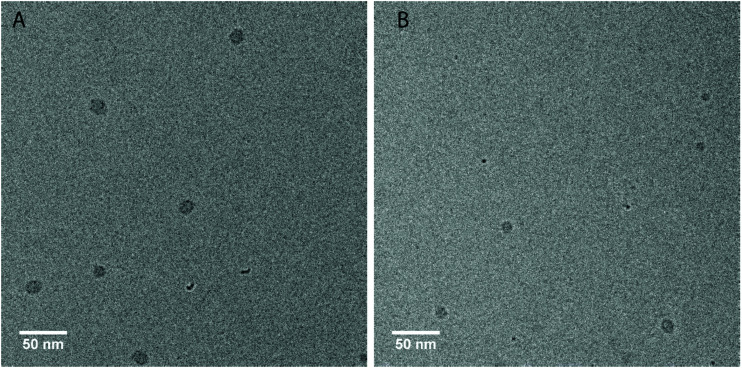
Cryo-TEM micrographs of **PUI-A2** (A) and **PUI-S2** (B) after vitrification from aqueous 0.1 M TEA solutions. The grey spots are the micelles vitrified in the ice layer, while the smaller black spots are ice-crystal artefacts at the surface. Concentration: approximately 2.5 mg mL^−1^. Magnification: 48 000. The ESI contains additional cryo-TEM micrographs with ESI.†

**Fig. 5:**
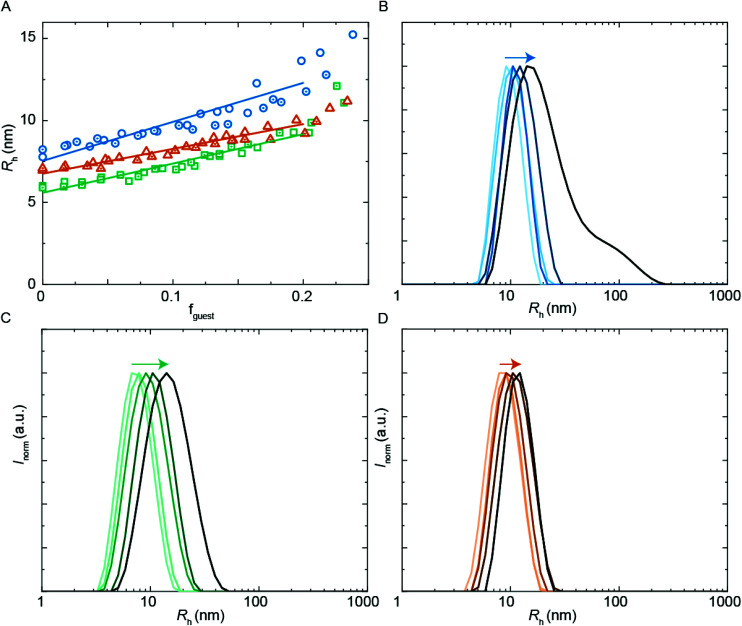
DLS encapsulation experiments using **PU-S0** as guest. (A) *R*_h_ from DLS as a function of guest loading for **PUI-A2** (blue circles), **PUI-S2** (green squares) and an equimolar mixture of these two hosts (orange triangles), main *R*_h_ from size distributions by volume. The (cumulative) host concentration is 2.5 mg mL^−1^ for all data points. (B)–(D) Size distributions by intensity from DLS; arrows indicating increasing guest load. (B) **PUI-A2** host, guest loading 0, 0.056, 0.10, 0.14, 0.20 (low to high guest loading in blue gradient). (C) **PUI-S2** host, guest loading 0, 0.045, 0.10, 0.14, 0.21 (low to high guest loading in green gradient). (D) Equimolar **PUI-A2** and **PUI-S2** host, guest loading 0, 0.048, 0.096, 0.15, 0.19 (low to high guest loading in orange gradient).

In first measurements we have investigated the two amphiphilic polymers **PUI-A2** and **PUI-S2**, because previously reported SCF computations have indicated that these type of PUIs presumably play an important role in particle formation and stabilization in WPUs prior to chain extension.^14^ Indeed, **PUI-A2** and **PUI-S2** form micelles above a threshold concentration in single-component aqueous solutions. With cryo-TEM we have been able to visualize these micelles (Fig. 4), with recorded radii of about 7.2 nm for **PUI-A2** and 5.2 nm for **PUI-S2**. The micellar dimensions were also determined by dynamic light scattering (DLS), which yielded similar hydrodynamic radii (*R*_h_) of approximately 8.0 nm and 6.0 nm for **PUI-A2** and **PUI-S2**, respectively (Fig. 5A, *y*-axis intercept). In an earlier study, the size difference was attributed to packing restraints of the bolaamphiphilic structure of **PUI-S2** when compared to **PUI-A2**.^17^ The latter PUI resembles a classical head-to-tail surfactant architecture, although **PUI-A2** is of course much larger in size than typical surfactants. In the following we refer to **PUI-A2** and **PUI-S2** as ‘hosts’, since their association colloids may solubilize and encapsulate additional compounds in their interior (core), and possibly also in their exterior (corona).

Next, the remaining three sequences **PU-S0**, **PUI-A1** and **sPUI-S1** have been briefly studied individually. These materials carry either a single charge (**sPUI-S1** and **PUI-A1**) or no charge at all (**PU-S0**). Unsurprisingly, the neutral and hydrophobic **PU-S0** is not soluble in a 0.1 M TEA solution in water. Rehydration of the **PUI-A1** thin film gives a turbid dispersion, that does not show precipitation. DLS measurements do not show micelle formation and only reveal ill-defined larger aggregates in the 100–1000 nm radius size range (intensity plot; data not shown). For **sPUI-S1**, that lacks a surfactant architecture, we find clear solutions in water that show large non-micellar aggregates, roughly 40 nm in radius (data not shown).

Complementary SCF computations were performed to obtain further insight into the micellization of the PUIs that contain both hydrophobic poly-THF and charged DMPA. Initial SCF computations predicted that molecules with two charges, *i.e.***PUI-A2** and **PUI-S2**, form micellar-type of assemblies, in line with our experimental findings (Fig. S6†). SCF computations on **PUI-A1** showed that this PUI type does not spontaneously micellize in water (Fig. S6†). This computation is in line with the observed turbidity and large aggregate sizes observed for **PUI-A1** in DLS. Apparently, the hydrophilic content of this sequence is too low for self-assembly into stable micelle hydrocolloids. SCF computations on **sPUI-S1** yield molecularly dissolved species, presumably due to thermodynamic equilibrium constraints (data not shown). This SCF prediction is in contrast to the non-micellar aggregates observed in DLS.

### Encapsulation of hydrophobic **PU-S0** guest

A common application of surfactant formulations is to encapsulate lipophilic compounds. Understanding the behavior in multicomponent systems and improving the encapsulation efficiency is key towards developing new product formulations. In this work, we have studied the size of assemblies when the hydrophobic **PU-S0** material was added as a ‘guest’ to the double-charged ‘host’ sequences. The poly-THF chains of the hosts should act in analogy to alkyl chains in typical surfactants, yielding micelles with a hydrophobic core, thus creating an environment capable of encapsulating the otherwise water-insoluble hydrophobic **PU-S0** guest. Recent work has shown that host structures can either stretch to accommodate a hydrophobic guest, or alternatively get expelled from the core and populate the exterior of the particles.^15^

We first studied the solubilization of the symmetric guest **PU-S0** (Fig. 5A) in micelles of either the asymmetric **PUI-A2** or the symmetric **PUI-S2**. We anticipated the neutral and hydrophobic guest to favor association, and to reside in the micellar core preferentially. Therefore, we expected that increasing the guest loading would increase the micellar size. Indeed, solubilization of the hydrophobic guest **PU-S0** increased the micellar dimensions markedly from *R*_h_ ≈ 8.0 nm and *R*_h_ ≈ 6.0 nm at *f*_guest_ = 0 for the pure host solutions up to *R*_h_ ≈ 12.7 nm and *R*_h_ ≈ 9.6 nm for mixed micelles of **PUI-A2** or **PUI-S2**, respectively, at a **PU-S0** loading *f*_guest_ ≈ 0.2. Hence, both micelle types grow approximately 1.6-fold in radius (so approximately 4.0-fold in volume) upon loading the micelle with about 20 w/w% guest molecules. As the host PUI concentration remains unchanged upon adding guests, with the micelles growing considerably, we assume that at a guest loading of *f*_guest_ ≈ 0.2 the number of micelles is reduced markedly (corresponding to an increased host aggregation number per micelle).

**Fig. 6:**
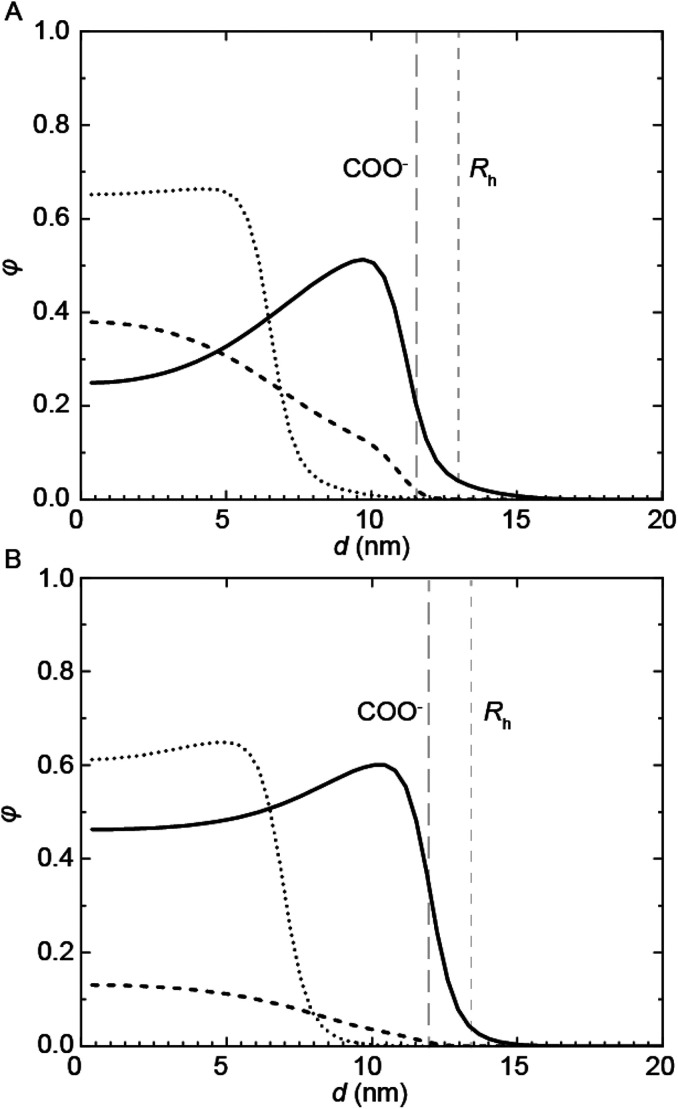
SCF computations showing the concentration profiles, *φ*, of (A) **PUI-A2** and (B) **PUI-S2** with (solid) and without (dotted) the presence of the **PU-S0** guest (dashed) as a function of the distance from the center of the micelle, d. The host profiles in presence of guest correspond to the maximum loading of *f*_guest,SCF_ = 0.224 for **PUI-A2** and 0.049 for **PUI-S2**. The COO^−^ group positions and the micellar radii of the loaded micelles are indicated in the graphs.

Complementary SCF computations were performed to obtain further insight into the micellization of the PU(I)s. SCF computations allow to determine the location of not only the individual components, but also of their building blocks, within mixed micelles. This is achieved by comparing the radial concentration profiles, *φ*, as a function of the distance from the center of the micelle, *d*, of the PUI hosts alone (referred to as ‘empty’) to the individual PU(I) sequences in the mixtures with **PU-S0** (Fig. 6). Not surprisingly, we found that the charged carboxylic acid-group of DMPA is mainly located near the outer surface of the micelle. In the mixtures, the radius (*R*_h_) is approximated by the *d*-value where *φ*_host_ approaches zero. In both empty micelle systems, the concentration distributions are fairly homogeneous throughout the micelle. The distribution of **PUI-S2** within the micelle remains similar upon adding **PU-S0** guest and only shifts to a larger *d*. This seems to indicate that the poly-THF chains of the **PUI-S2** host stretch to accommodate **PU-S0**. Interestingly, the shape of the **PUI-A2** host profile upon addition of **PU-S0** shows a stark change as compared to that of the host alone, with the **PU-S0** concentration in the core even exceeding the concentration of poly-THF chains from the host. This pinpoints that **PUI-A2** is partially expelled from the center of the micelle and substituted with **PU-S0** guest. We attribute the difference in host and guest distribution along the micelle to the bolaamphiphilic structure of **PUI-S2**, which structure limits the degree of freedom it has to rearrange and accommodate guest compounds. In comparison, **PUI-A2** has a dangling poly-THF chain that can more freely move and rearrange. In the computations, this difference is reflected in the theoretical maximum encapsulation efficiencies as well, as the maximum attainable *f*_guest,SCF_ before divergence occurs equals 0.049 for **PUI-S2** and 0.224 for **PUI-A2**. These calculations therefore indicate that the bolaamphiphile architecture of **PUI-S2** restricts the amount of hydrophobic guests that can be accommodated, whereas **PUI-A2** is able to encapsulate a higher loading of guest. This discrepancy between the encapsulation efficiencies of the two hosts as predicted with SCF was not observed experimentally, as both **PUI-A2** and **PUI-S2** accomodated **PU-S0** comparably well.

Having established the efficient encapsulation of the hydrophobic guest **PU-S0** in PUI micelles of either **PUI-S2** or **PUI-A2**, we subsequently focused on the ternary system wherein the **PUI-S2** and **PUI-A2** hosts were mixed at an equimolar ratio. The size of the mixed micelles remained intermediate between that of either of the constituent PUI hosts up to *f*_guest_ ≈ 0.2, which indicates molecular mixing of both hosts.^20^ The mixed micelles grew approximately 1.4-fold in size from an *R*_h_ of 7.0 nm in the absence of **PU-S0** to *R*_h_ ≈ 9.6 nm at *f*_guest_ ≈ 0.2, corresponding to about a 2.6-fold growth in volume. The increase in micellar size at the same **PU-S0** loading is thus smaller for the mixed PUI micelle as compared to micelles composed of either the **PUI-A2** or the **PUI-S2** host. This may be due to the increased configurational freedom within the mixed core: the **PUI-S2** chains might relax in this morphology compared to their energetically unfavorable stretched state in the single host micelle. This clarification is reminiscent and in line with recent findings on a binary diblock mixture differing in core block length.^21^ Interestingly, when comparing the size distributions by intensity for guest fractions *f*_guest_ from 0 progressively to 0.2, we observe broadening for the individual **PUI-A2** (Fig. 5B) and **PUI-S2** (Fig. 5C) host micelles, whereas the equimolar host mixture (Fig. 5D) yields a narrow distribution for all tested *f*_guest_ values. Additionally, at higher guest loading for the **PUI-A2** host, we observe a broad shoulder in the size distribution (Fig. 5B), indicating the onset of formation of larger ill-defined (non-micellar) aggregates, which signals that these samples are approaching the stability boundary. The data suggest a somewhat enhanced encapsulation efficiency and an improved colloidal micellar stability for the ternary system as compared to the binary ones. This is in line with observations made for a number of mixed polymeric systems reviewed by Attia and coworkers,^22^ and for mixed surfactant micelles.^23,24^ In future studies, it would be of interest to further optimize the composition of PU(I) mixtures to increase the solid content; for example with regard to coating applications, thereby improving drying processes and decreasing costs.^24^

### Encapsulation of the single-charged hydrophilic **sPUI-S1** guest

**Fig. 7:**
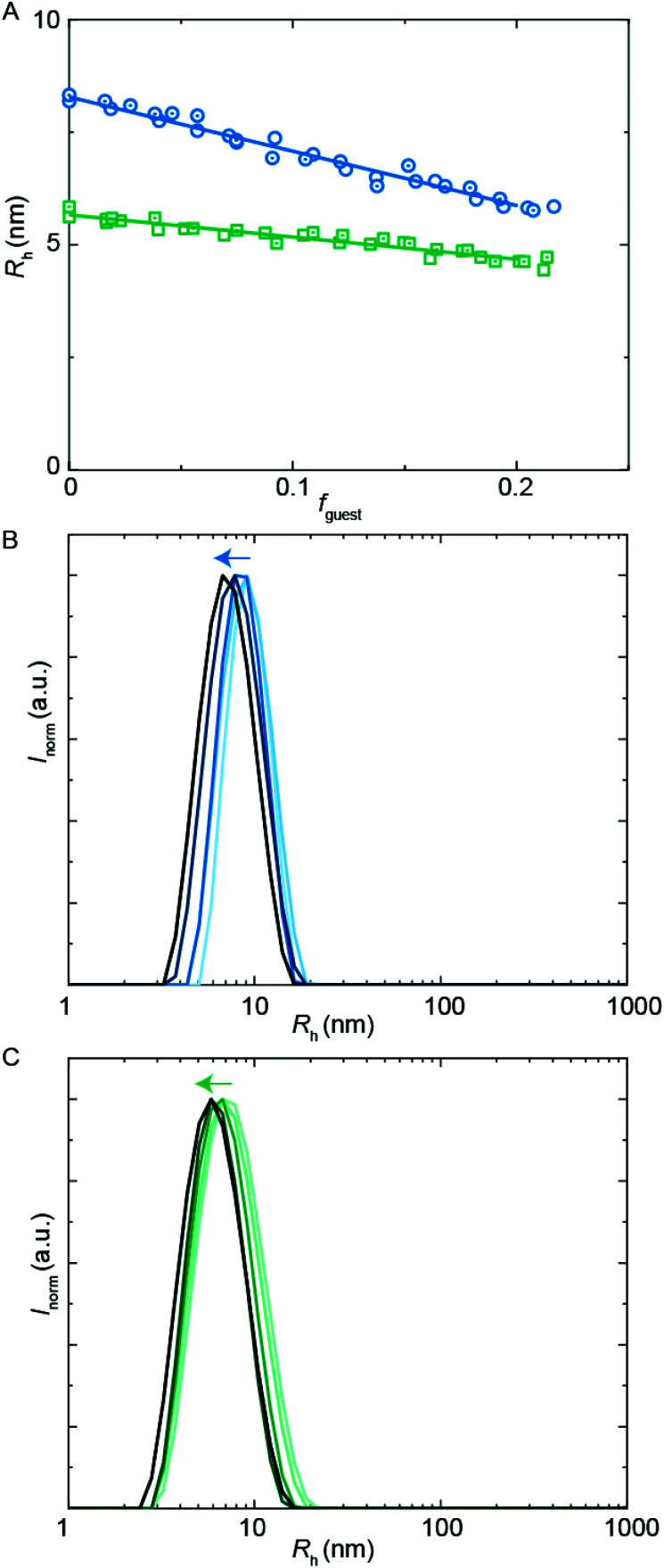
DLS encapsulation experiments using **sPUI-S1** as guest. (A) *R*_h_ as a function of guest loading for the **PUI-A2** (circles) and **PUI-S2** (squares) hosts, main *R*_h_ from size distributions by volume. The host concentration is 2.5 mg mL^−1^ for all data points. (B) and (C) Size distributions by intensity from DLS; arrows indicating increasing guest load. (B) **PUI-A2** host, guest loading 0, 0.057, 0.092, 0.15, 0.20 (low to high guest loading in blue gradient). (C) **PUI-S2** host, guest loading 0, 0.052, 0.10, 0.15, 0.20 (low to high guest loading in green gradient).

Besides hydrophobic molecules, commercial WPU formulations prior to chain extension (*vide supra*) additionally contain small molecules such as **sPUI-S1**.^14^ This small and symmetric **sPUI-S1** guest is not likely to reside in the micelle core since it is charged, and instead it may prefer the periphery of the micelle. This could manifest^15^ as a decrease in micellar dimensions upon an increase in **sPUI-S1** loading, since **sPUI-S1** in this scenario would predominantly contribute to the repulsive interactions between the charged segments within the micellar shell. We indeed observed in DLS experiments that both **PUI-S2** and **PUI-A2** micelles decrease in size upon an increase in the **sPUI-S1** guest concentration (Fig. 7A). When comparing *f*_guest_ = 0 to *f*_guest_ ≈ 0.2, we observe a decrease from an *R*_h_ of 5.7 nm to ≈4.6 nm (roughly a decrease of 20% in radius, and a 2-fold decrease in volume) for **PUI-S2** and from 8.3 nm to ≈5.8 nm (roughly a decrease of 30% in radius, and a 3-fold decrease in volume) for **PUI-A2**, respectively. The decreasing micelle size is in line with previous work by Ianiro *et al.*^15^ It is worth noting that the size distributions remain narrow at higher guest loadings (Fig. 7B and C).

**Fig. 8:**
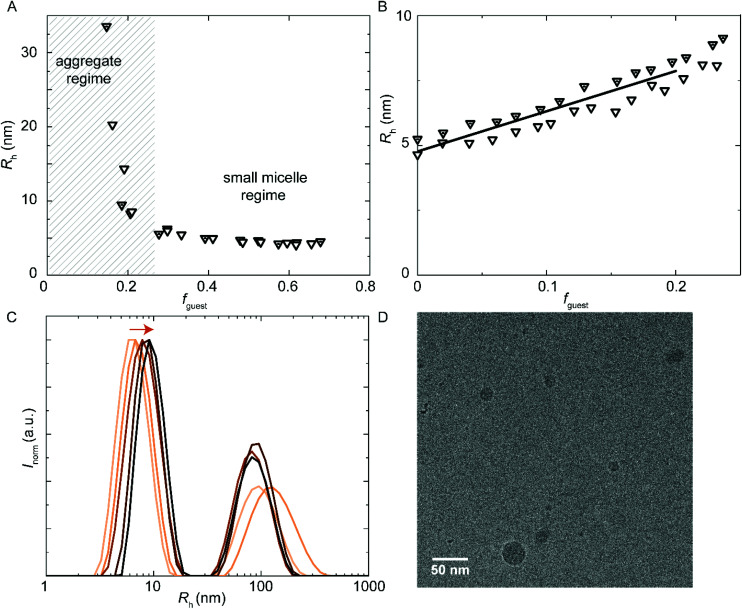
DLS encapsulation experiments. (A) Addition of **sPUI-S1** guest to **PUI-A1** host that does not micellize on its own. At **sPUI-S1***f*_guest_ > 0.27, micelle formation is induced. (A and B) Main *R*_h_ from size distributions by volume. The (cumulative) host concentration is 2.5 mg mL^−1^ for all data points. (B and C) Encapsulation of **PU-S0** guest into **PUI-A1** : **sPUI-S1** (1 : 6 molar ratio host) co-micelles. (B) *R*_h_ as a function of **PU-S0** guest loading. (C) Size distributions by intensity from DLS, arrow indicating increasing guest load, at guest loading 0, 0.041, 0.095, 0.15, 0.20 (low to high guest loading in orange gradient). (D) CryoTEM micrograph of **PUI-A1** : **sPUI-S1** (1 : 6 – mol : mol) host with added **PU-S0** guest *f*_guest_ ≈ 0.2. Cumulative host concentration: approximately 2.5 mg mL^−1^. Magnification: 48 000. The ESI contains additional cryo-TEM micrographs with ESI.†

Since **sPUI-S1** augments the repulsive double layer interactions which enhance colloidal stability at low ionic strength in the above binary systems, we next studied whether **sPUI-S1** could prompt micellization of a PUI component that is incapable of forming micelles on its own, *i.e.***PUI-A1**. We first added increasing amounts of **sPUI-S1** to **PUI-A1** and monitored the turbidity of the solutions, thereby aiming at formation of stable hydrocolloids with dimensions similar to those found for our other hosts (Fig. 8A). At *f*_guest_ < 0.27, the mixtures were turbid dispersions containing aggregates with sizes much larger than 10 nm. Interestingly, at *f*_guest_ > 0.27, the samples became transparent and the main size distributions correspond to *R*_h_ ≈ 5.8 nm (Fig. 8A), which is comparable to the micellar sizes obtained with **PUI-S2** or **PUI-A2**. Hence, *f*_guest_ ≈ 0.27 demarcates the threshold between two regimes: the large aggregate regime and the small mixed-micelle regime. Accordingly, we have found a synergy between **sPUI-S1** (that has a high charge-to-weight ratio, but lacks a surfactant architecture) and **PUI-A1** (that has a head-to-tail surfactant molecular structure, but that is lacking in charge), as only together they form stable micelles.

Further, and to assess the encapsulation efficiency of the prepared mixed micelles of **PUI-A1** and **sPUI-S1**, we have studied their size and stability upon addition of **PU-S0** guest. For this purpose, we have employed mixed micelle hosts containing **PUI-A1** and s**PUI-S1** in a 1 : 6 molar ratio (this corresponds to *f*_guest_ = 0.62 in Fig. 8A). In analogy to the other PU(I) micelles described in this work, *R*_h_ increased with the **PU-S0** guest concentration from 4.9 nm at *f*_guest_ = 0 to *R*_h_ ≈ 7.9 nm at *f*_guest_ ≈ 0.2, displaying a 1.6-fold increase in radius, corresponding to about a 4-fold increase in volume (Fig. 8B). Note that in this ternary system a minor fraction of the PU(I)s remains incorporated in large aggregates, indicated by the bimodal size distributions at all guest loadings (Fig. 8C). However, as the intensity scales with *R*^3^, the secondary small peak represents a very small fraction of scatterers. Hence, the micelles are in all cases the most abundant species. Again, this ternary system shows synergy as none of the three components is capable of forming micelles individually, but the ensemble of molecules produces stable micelles with hydrophobic guest encapsulated. Finally, cryo-TEM measurements confirmed increased dimensions for **PUI-A1**/**sPUI-S1** micelle hosts upon incorporation of **PU-S0** guest (Fig. 8D; Fig. S9 and S10†), showing radii of 4.0 and 7.1 nm for micelles without and with **PU-S0** guest (*f*_guest_ ≈ 0.2), respectively. As also seen for **PUI-A2** and **PUI-S2** micelles (Fig. 4 and 5), we observe a good agreement between radii assessed with DLS and cryo-TEM.

On a final note, the **sPUI-S1** sequence can be viewed as quite a good model for IPDI-DMPA-IPDI molecules that are abundant in WPU formulations prior to the chain extension reaction (*vide supra*), provided of course that both isophorone di-isocyanate (IPDI) and DMPA building blocks are employed. The experimental data in this study show that **sPUI-S1** behaves as an effective co-surfactant, promoting stable mixed-micelle formation. Presumably, therefore, the actual IPDI-DMPA-IPDI molecule in industrial WPUs plays a similar role. It stabilises the WPU dispersion prior to chain extension, but probably also during and after the chain extension reaction: it is likely positioned at the periphery of particles prior to chain extension, and it presumably remains there as it gets trapped during the chain-extension reaction that results in a diminished mobility of molecular chains. This role of **sPUI-S1** is not apparent from our SCF computations, nor from those reported on in literature,^14^ highlighting the importance of the combination of computational and experimental research in this field. Indeed, experimental results may serve as an incentive to improve SCF models, for example such that the role of **sPUI-S1** will become better captured by SCF computations.

## Conclusions

**Fig. 9:**
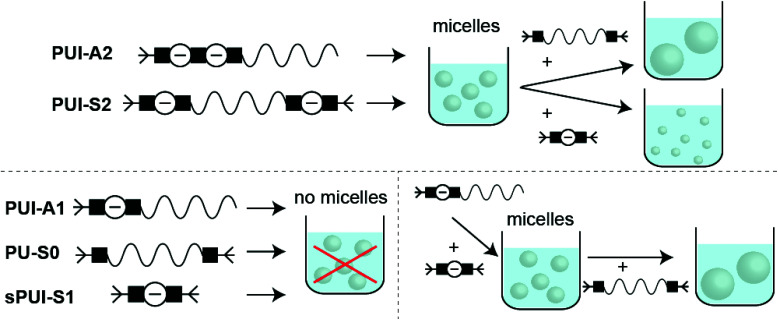
Overview of monomeric PU systems, or binary and ternary PU mixtures in water, leading to micelles, no micelles, or mixed-micelles. The growth or shrinkage of co-micelles due to incorporation of guests is also represented.

For this paper we have studied the assembly of macromolecules in multicomponent amphiphilic polyurethane systems (Fig. 9). First, and in a novel synthetic approach to preparing precision PUs, we have successfully produced five sequence-defined polyurethanes: asymmetric polyurethane ionomers with one (**PUI-A1**) or two (**PUI-A2**) charges; a symmetric PUI with two charges (**PUI-S2**); a symmetric, neutral and hydrophobic polymer (**PU-S0**) and a small and symmetric ionomer with one charge (**sPUI-S1**). We have applied scattering experiments (DLS), SCF-computations and cryo-TEM measurements to unravel the behavior in water of individual PU(I) sequences as well as of mixtures (of exactly known composition) of PU(I) sequences. This strategy revealed that hydrophobic **PU-S0** is encapsulated in micelle-forming sequences, thereby increasing micellar dimensions. Particularly, we have found that **PUI-S2** micelles are slightly distorted by encapsulation as the host chains stretch to accommodate the guest, whereas **PUI-A2** is partially expelled from the core due to its ability to rearrange more easily. The ternary mixture of **PUI-A2** and **PUI-S2** hosts and **PU-S0** guest displayed improved micellar stability as compared to single host dispersions. Additionally, the small **sPUI-S1** guest caused a reduction of the micellar size by augmenting the repulsion within the micellar shell. Interestingly, **sPUI-S1** when mixed with **PUI-A1** acts as a co-surfactant, as co-micelles of these components are capable of encapsulating hydrophobic **PU-S0** within stable micellar hydrocolloids. Note that none of these three components efficiently forms micelles on their own, signposting a synergistic ensemble effect in this ternary PU(I) system. All results considered, our approach of preparing sequence-defined PUs and studying them individually as well as in mixtures of exactly known composition provides a viable method to extract information on complex multi-components mixtures. We also believe that this strategy can be extended to other polymeric mixtures relevant for food, pharmaceutical, coating and other industries.

## Author contributions

Conceptualization EMT, AGG, ReT, HMJ, IKV; data curation EMT, PMF, AGG, SMCS, JWP; formal analysis EMT, PMF, AGG, SMCS; funding acquisition ReT, HMJ, IKV; investigation EMT, PMF, AGG, SMCS, JWP, HMJ; methodology EMT, PMF, AGG, HMJ, ReT; project administration RoT, ReT, HMJ, IKV; resources PMV, HMJ; Software (Malvern Zetasizer 6.12; ImageJ; SFbox); supervision ReT, HMJ, IKV; validation EMT, AGG, PMF, JRM, ReT, HMJ, IKV; visualization EMT, PMF, AGG, SMCS, JRM; writing – original draft EMT, AGG, JRM, HMJ; writing – review & editing EMT, AGG, SMCS, JRM, RoT, IC, ReT, HMJ, IKV.

## Conflicts of interest

There are no conflicts to declare.

## Supplementary Material

PY-012-D1PY00079A-s001
