# Supplementary material for: Co-assembly of precision polyurethane ionomers reveals role of and interplay between individual components
Source: Polym Chem. 2021 Apr 22;12(19):2891–903. doi: 10.1039/d1py00079a (PMC8129887; doi:10.1039/d1py00079a)

Electronic Supplementary Material (ESI) for Polymer Chemistry.
This journal is © The Royal Society of Chemistry 2021

Pioneering Investigator 2021: Co-assembly of precision polyurethane ionomers reveals role of and interplay between individual components

Elizabeth M. Timmers[†,‡,¶], *P. Michel Fransen*[§], *Álvaro González García*[‖,¶,⊥], *Sandra M. C. Schoenmakers*[‡,¶], *Jose Rodrigo Magana*[†,‡,¶], *Joris W. Peeters*[§], *Ronald Tennebroek*[⁋], *Ilse van Casteren*[⁋], *Remco Tuinier*[‖,¶,⊥], *Henk M. Janssen*[§,⁑] *and Ilja K. Voets**[†,‡,¶,‖,⁑]

† Laboratory of Self-Organizing Soft Matter, Department of Chemical Engineering and Chemistry, Eindhoven University of Technology, P.O. Box 513, 5600 MB, Eindhoven, The Netherlands

‡ Laboratory of Macro-Organic Chemistry, Department of Chemical Engineering and Chemistry, Eindhoven University of Technology, P.O. Box 513, 5600 MB, Eindhoven, The Netherlands

¶ Institute for Complex Molecular Systems, Eindhoven University of Technology, P.O. Box 513, 5600 MB, Eindhoven, The Netherlands

§ SyMO-Chem B.V., Den Dolech 2, 5612 AZ, Eindhoven, The Netherlands

‖ Laboratory of Physical Chemistry, Department of Chemical Engineering and Chemistry, Eindhoven University of Technology, P.O. Box 513, 5600 MB, Eindhoven, The Netherlands

⊥ Van 't Hoff Laboratory for Physical and Colloid Chemistry, Department of Chemistry and Debye Institute for Nanomaterials Science, Utrecht University, Padualaan 8, 3584 CH, Utrecht, The Netherlands

⁋ DSM Resins & Functional Materials , Sluisweg 12, 5145 PE Waalwijk, the Netherlands

* Corresponding author

⁑ **These authors contributed equally**

Electronic Supplementary Information

ESI Sections

1. Molecular structures of the sequence-defined precision PU(I) materials
2. The synthesis and molecular characterization of the sequence-defined PU(I)s
3. SCF information
4. Cryo-TEM analyses
5. DSC information
6. References
7. NMR and IR spectra, DSC thermograms of **sPUI-S1**, **PU-S0**, **PUI-A1**, **PUI-A2**, **PUI-S2**

1. Molecular structures of the sequence-defined precision PU(I) materials

Scheme S1 shows the (macro)molecular structures of the PU(I)s central to this study.

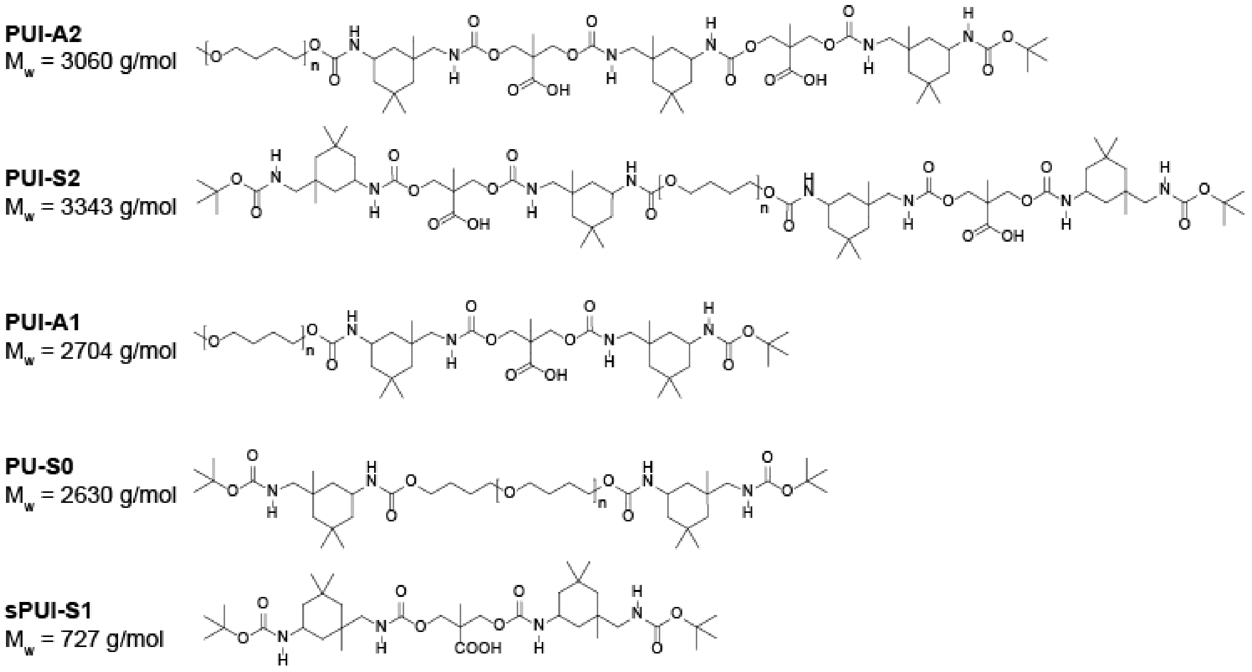

Scheme S1: Molecular structures of the PU(I) materials as used in this study. The average molar mass of pTHF is about 2 kDa. Given molecular weight values are for pTHF chains with 28 THF-units. Note that the IPDA building blocks exist in multiple regio- and stereoisomers; this is not shown for brevity.

2. The synthesis and molecular characterization of the sequence-defined PU(I)s

A. Overview

B. Sequence-defined precision versus purity versus mono-dispersity

C. Materials, molecular characterization and abbreviations

D. The synthesis of **sPUI-S1** including analytical data

E. The synthesis of **PUI-A1** including analytical data

F. Compiled analytical data on **PU-S0**, **PUI-S2** and **PUI-A2**

G. Analytical data on the sequence-defined PU(I)s: MS, SEC and NMR figures and tables

A. Overview

The syntheses of the symmetrical **PU-S0** and **PUI-S2** materials, the asymmetrical **PUI-A2** material and the building blocks **BB** and **5** have been described extensively in recent work (see reference 1). Materials **1**, **2**, **3** and **6** have been described there as well, but have been highlighted here once more to properly show the synthetic origin of newly presented materials **sPUI-S1** and **PUI-A1** that both are described herein in detail. The synthetic routes to these two mono-charged materials are shown in Scheme S2 and S3.

Analytical data on all five PU(I)s are included in sections D to G.

B. Sequence-defined precision versus purity versus mono-dispersity

The presented organic synthetic approach to the PU(I)s allowed for the build-up of macromolecules with a strictly defined order of components, i.e. macromolecules with a sequence-defined microstructure. In the prepared precision materials, the number of IPDA, DMPA and pTHF groups is precisely controlled, as well as the positioning of these groups within the macromolecular structure. The produced PU(I)s are not chemically pure, though, where this is due to (i) the used IPDA component (introducing isomeric diversity) and (ii) the employed poly-THF component (introducing a distribution and dispersity in molecular weight). Accordingly, the produced materials are also not monodisperse (apart from **sPUI-S1**), as all macromolecules would then have to be of the same molecular weight.

Indeed, all presented and prepared PU(I) products in this paper are composed of a complex mixture of regio- and stereo-isomers. This is due to the employed IPDA reactant that as a building block is already a mixture of stereo- and diastereo-isomers. Moreover, IPDA can react at both amine groups with Boc-anhydride, so amine **1** becomes a mixture of two regiomers (see Scheme S2). Of

course, all molecules **1** have only one amine and only one Boc-protected amine group, so in this sense amine **1** is defined. Similarly, building block **BB** (Scheme S3) is molecularly defined as it has only one active carbonate group, only one benzyl-ester group and only one Boc-protected amine group.

We have chosen the isophorone diamine (IPDA) building block for our syntheses, as commercial waterborne polyurethanes are very frequently prepared from isophorone diisocyanate (IPDI). We have considered using hexane-diamine/diisocyanate (HDA/HDI) building blocks instead, which would have resulted in materials with more regular molecular structures and more regularly positioned hydrogen bonds. However, and importantly, this could have easily led to materials that are decidedly more crystalline in nature. Indeed, a comparative study on polyester thermoplastic elastomers, using either HDI or IPDI building blocks, shows clear crystalline phases for only the HDI-material (reference 2). In designing the sequence-defined PU(I)s of this study, we did not want to introduce a factor (i.e. crystallinity) that could dominate the behavior of these materials, given that this factor is not important in industrially employed IPDI-based WPUs. In conclusion, we wanted the designed and prepared sequence-defined PU(I) to reflect the properties of industrial WPUs as close as possible.

C. Materials, molecular characterization and abbreviations

Materials

All reagents, chemicals, materials and solvents were obtained from commercial sources, and were used as received: Cambridge Isotope Laboratories for (deuterated) solvents, Aldrich, Acros, ABCR, Merck and Fluka for chemicals, materials and reagents. All solvents were of AR quality. Moisture or oxygen-sensitive reactions were performed under an atmosphere of dry argon. Analytical thin layer chromatography was performed on Kieselgel F-254 precoated silica plates. Column chromatography was carried out on Screening Devices B.V. flashsilica gel (40-63 μm mesh) or normal silica gel (60-200 μm mesh). In the polymer extension reactions, and for scavenging the excess of **BB** building block, an amine terminated resin was employed (Silicycle Si-Amine catnr. R5203B loading 1.89 mmol/g). For hydrogenations 10% Pd/C Degussa type E101 NE/W (Sigma-Aldrich) was used.

Molecular characterization

NMR spectra were recorded on a 400 MHz Varian Mercury spectrometer at 298 K. Chemical shifts are reported in ppm downfield from TMS at room temperature using deuterated chloroform ($CDCl_3$) as a solvent and internal standard unless otherwise indicated. Abbreviations used for splitting patterns are s = singlet, t = triplet, q = quartet, m = multiplet (or multiple signals), dd =

double doublet, and b or br = broad. Matrix-assisted laser desorption ionization time-of-flight mass spectrometry (MALDI-TOF MS) was performed on a PerSeptive Biosystems Voyager-DE PRO spectrometer. As indicated, these measurements were done by using a α-cyano-4-hydroxycinnamic acid (CHCA) matrix, where KAc was optionally added to the matrix to primarily generate K^+-adducts, and a linear positive mode was employed. Size-exclusion chromatography (SEC) was measured on a Shimadzu LC-10AD VP system with a RID-10A detector and a SPD-M20A diode array detector using a PL gel 5μm mixed-C and a mixed-D column in sequence and using THF as the eluent (flow rate 1 mL min^{-1}). The SEC molecular weight and distribution data were recorded relative to polystyrene (PS) standards using refractive index (RI) detection. Attenuated total reflection Fourier-transform infrared (ATR-FT-IR) spectra were recorded on a Shimadzu IRAffinity spectrometer using MIRacle-10 ATR accessory. HPLC-PDA/ESI-MS was performed using a Shimadzu LC-10 AD VP series HPLC coupled to a diode array detector (Finnigan Surveyor PDA Plus detector, Thermo Electron Corporation) and an Ion-Trap (LCQ Fleet, Thermo Scientific). Electrospray ionization (ESI) was used to create charged species for mass detection. Analyses were performed at 298 K using an Alltech Alltima HP C18 3μ column using an injection volume of 1-4 μL, a flow rate of 0.2 mL min^{-1} and typically a gradient (5% to 100% in 10 min, held at 100% for a further 3 min) of CH_3CN in H_2O with both these eluents containing 0.1% formic acid.

Abbreviations

Tetramethylsilane (TMS), *N*,*N*-dimethylformamide (DMF), tetrahydrofuran (THF), dichloromethane (DCM), trifluoroacetic acid (TFA), benzyl (Bn), potassium hydroxide (KOH), size-exclusion chromatography (SEC), nuclear magnetic resonance (NMR), matrix-assisted laser desorption ionization time-of-flight mass spectrometry (MALDI-TOF MS), high performance liquid chromatography-photo diode array/electrospray ionization mass spectrometry (HPLC-PDA/ESI-MS), total ion current (TIC), attenuated total reflection Fourier-transform infrared (ATR-FT-IR).

D. The synthesis of sPUI-S1 including analytical data

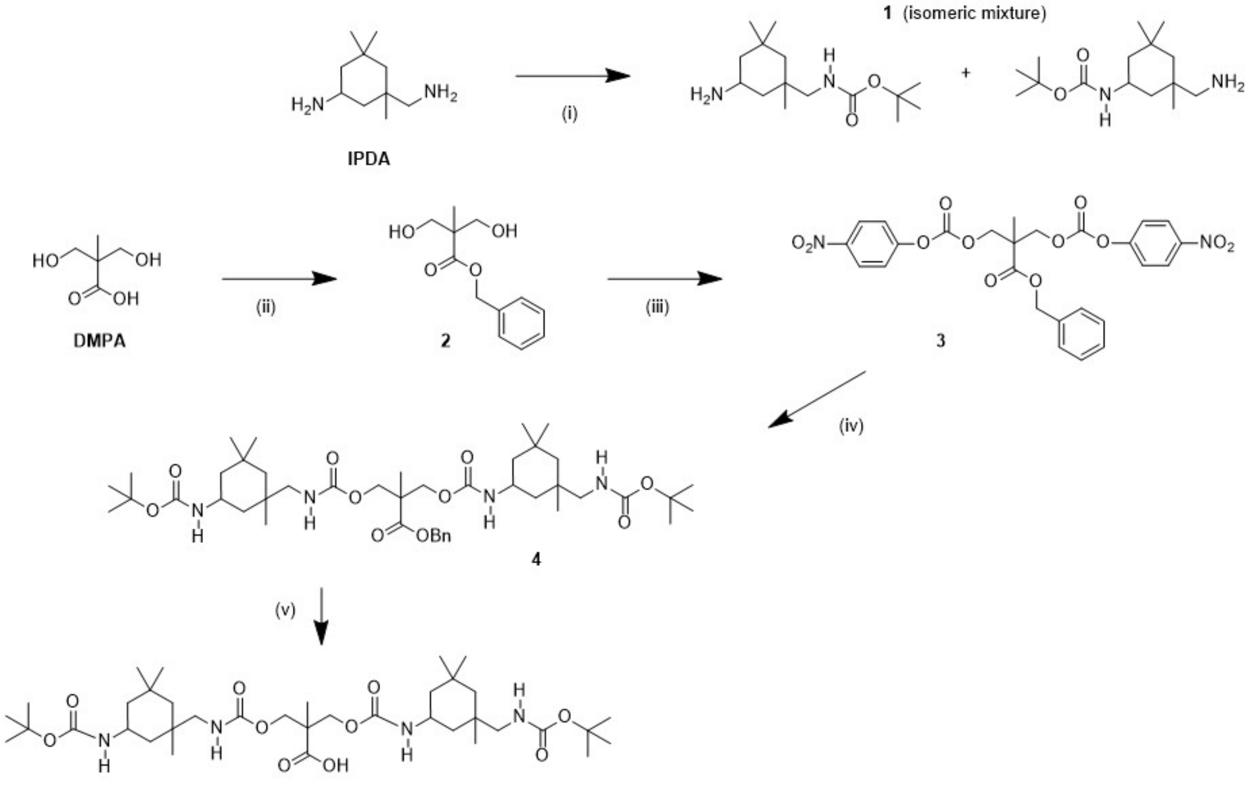

sPUI-S1

*Scheme S2: Synthesis route to small polyurethane ionomer **sPUI-S1**. (i) Boc-anhydride, DCM; (ii) BnBr, KOH, DMF, 100^{0}C; (iii) 4-nitrophenyl chloroformate, pyridine, DCM; (iv) amine **1**, pyridine, dioxane, reflux; (v) Pd/C, H_2, iso-propanol. Note that the IPDA building blocks exist in multiple regio- and stereoisomers; this is not shown for brevity.*

Isophorone diamine (IPDA) was reacted with Boc-anhydride to give, after purification, the mono-Boc-protected mono-amine **1** (Scheme S2). Di-methylol-propionic acid (DMPA) was protected with a benzyl-(Bn)-ester group and then activated using 4-nitrophenyl chloroformate to prepare the di-activated di-carbonate **3**. Molecule **3** was reacted with mono-amine **1** to produce the symmetrical benzyl-protected intermediate **4** that only required hydrogenation to afford **sPUI-S1**.

Mono-Boc isophorone diamine (Boc-IPDA-NH_2): isomeric mixture of tert-butyl [3-(aminomethyl)-3,5,5-trimethylcyclohexyl]carbamate and tert-butyl [(5-amino-1,3,3-trimethylcyclohexyl)methyl]carbamate (molecule 1)

A solution of isophorone diamine (IPDA; 3 g, 17.6 mmol, 2 molar equivalents) in DCM (20 mL) was stirred at -78^{0}C. Boc-anhydride (1.92 g, 8.8 mmol) in DCM (80 mL) was added drop wise. After addition the mixture was allowed to heat up to room temperature, upon which it became hazy. HPLC-MS analysis showed that about 75% of mono-Boc product and 23% of di-Boc product had formed. The reaction mixture was evaporated, a solution of 0.1M formic acid (pH=3) was added, and this solution was washed two times with DCM (2 x 20 mL) to remove di-Boc product. The water layer was then brought to pH=9 with a 0.1M NaOH solution, and the product was extracted using two portions of DCM (20 mL). The combined organic layers were repeatedly washed with a solution of borax buffer (pH 9.3; 9 mL 0.1M NaOH and 91 mL 0.05M sodium tetraborate), until non-functionalized IPDA had disappeared from the organic layer. The presence of IPDA was checked by HPLC-MS analysis on a small sample after reaction with phenyl isocyanate. The organic layer was dried with Na_2SO_4 and concentrated to obtain the product as a waxy solid. Yield 3.6 grams (76%).

^{1}H NMR (400 MHz, chloroform-*d*) δ 4.60 (N*H*, m, 1H), 3.19 – 2.61 (C*H*N and C*H*$_2$N, m, 3H), 1.82 – 1.57 (CC*H*$_2$C, m, 2H), 1.55 – 1.40 ((C*H*$_3$)$_3$, s, 9H), 1.40 – 0.59 (N*H*$_2$, CC*H*$_2$C and C*H*$_3$C, m, 15H). HPLC-MS: 4.80 minutes, single peak, [M+H$^+$] = 271.08 *m/z*. Calculated: $C_{15}H_{30}N_2O_2$ (exact 270.23 g/mol; molar mass 270.42 g/mol).

DMPA(Bn)-diol: benzyl 3-hydroxy-2-(hydroxymethyl)-2-methylpropanoate (molecule 2)

Molecule **2** has previously been reported in reference 3.

Di-methylol-propionic acid (DMPA, 100 g, 746 mmol) and KOH (48.2 g, 732 mmol) were stirred in DMF (500 mL) for one hour at 100^{0}C. Benzyl bromide (153.4 g, 890 mmol, 1.2 molar equivalents) was added dropwise to the hot mixture that was thereafter stirred overnight at 100^{0}C under argon. A KBr-suspension formed. The mixture was concentrated to dryness by evaporation of the volatiles. The crude product was dissolved in a 1/1 mixture of ethyl acetate and hexane (1L), and this solution was washed with several portions of water (2L total volume). The organic layer was dried with Na_2SO_4, filtered and the filtrate was concentrated. The residue was recrystallized from toluene (300 mL) to yield a first crop of 96.6 g (58%) of product. The filtrate was concentrated and once more recrystallized to give a second crop (6.3 grams).

^{1}H NMR (400 MHz, chloroform-*d*) δ 7.34 (Ar-*H*, m, 5H), 5.18 (C*H*$_2$Bn, s, 2H), 3.90 – 3.70 (C*H*$_2$O, dd, 4H), 3.3 (O*H*, b, 2H), 1.09 (C*H*$_3$, s, 3H). HPLC-MS: 4.55 minutes, single peak, [M+H$^+$] = 225.00 *m/z*. Calculated: $C_{12}H_{16}O_4$ (exact 224.10 g/mol; molar mass 224.26 g/mol).

DMPA(Bn) di-(4-nitrophenyl carbonate): benzyl 2-methyl-3-(((4-nitrophenoxy)carbonyl)oxy)-2-((((4-nitrophenoxy)carbonyl)oxy)methyl)propanoate (molecule 3)

Molecule **3** has previously been reported in reference 3.

Diol **2** (3 g, 13.3 mmol) and 4-nitrophenyl chloroformate (5.92 g, 29.3 mmol, 2.2 molar equivalents) and pyridine (2.1 mL, 26.6 mmol, 2 molar equivalents) were dissolved in DCM (80 mL). The reaction mixture was stirred at room temperature for 1 hour; completion of the reaction was monitored by HPLC-MS and ^{1}H-NMR analysis. The mixture was washed with two portions of $NaHSO_4$ solution and thereafter with a $NaHCO_3$ (or a 0.1M NaOH) solution to remove 4-nitrophenol. Finally, the organic layer was dried with Na_2SO_4 and concentrated. The crude product was purified with column silica chromatography, first eluting with chloroform to remove traces of 4-nitrophenol and then eluting with 7v/v% ethyl acetate in chloroform to collect the product. Yield 4.17 g (56%).

^{1}H NMR (400 MHz, chloroform-*d*) δ 8.26 (Ar-*H*, m, 4H), 7.41 – 7.29 (Ar-*H*, m, 9H), 5.24 (CH_2-Bn, s, 2H), 4.68 – 4.42 (CH_2O, m, 4H), 1.41 (CH_3, s, 3H). HPLC-MS/PDA: 7.60 minutes, single peak (only PDA signal; no MS signal). Calculated: $C_{26}H_{22}N_2O_{12}$ (exact 554.12 g/mol; molar mass 554.46 g/mol).

Boc-IPDA-DMPA(Bn)-IPDA-Boc (molecule 4)

Di-carbonate **3** (1.0 gram, 1.81 mmol) was mixed with Boc-IPDA-NH_2 (amine **1**, 1.07 gram, 3.97 mmol, 2.2 molar equivalents) and pyridine (0.32 mL, 3.97 mmol) in dioxane (10 mL). The reaction mixture was stirred overnight at an oil bath temperature of 115^{0}C. Solvents were evaporated and the residue was dissolved in chloroform. The organic solution was washed with portions of a 0.1M NaOH solution until the water layer was colorless and not yellow anymore due to the presence of 4-nitro-phenolate, and was then consecutively washed with saturated NaCl (aq), a 0.025M HCl solution and finally with saturated NaCl (aq). The chloroform solution was dried with Na_2SO_4 and then concentrated. The residue, a yellowish product, was further purified by silica column chromatography applying a 5 v/v% MeOH in $CHCl_3$ eluent. Yield 1.19 gram (81%).

^{1}H NMR (400 MHz, chloroform-d) δ 7.33 (Ar-*H*, m, 5H), 5.16 (CH_2Ph, 2H), 4.88 – 4.32 (N*H*, m, 4H), 4.20 (CH_2OCON, br, 4H), 3.73 – 3.31 and 2.98 – 2.63 (C*H*N and CH_2N, m, 6H), 1.66 (CCH_2C, m, 4H), 1.44 ($(CH_3)_3$, s, 18H), 1.34 – 0.64 (CCH_2C and CH_3C, m, 29H). HPLC-MS: 9.52 minutes, two peaks for isomers, [M+Na^{+}] = 839.50 *m/z*. Calculated: $C_{44}H_{72}N_4O_{10}$ (exact 816.52 g/mol; molar mass 817.08 g/mol).

Boc-IPDA-DMPA-IPDA-Boc ("sPUI-S1")

Benzyl protected molecule **4** (1.19 gram, 1.46 mmol) was dissolved in iso-propanol (50 mL) and Pd/C Degussa type catalyst (100 mg). The mixture was shaken overnight in a Parr reactor with hydrogen gas (62 Psi). The mixture was filtrated over zeolite and the filtrate was concentrated by evaporation of the solvents yielding the product as an off-white solid. Yield: 1.00 gram (95%).

^{1}H NMR (400 MHz, chloroform-d) δ 5.87 (COO*H*, s, 1H), 4.88 – 4.54 (N*H*, m, 4H), 4.20 (CH_2OCON, b, 4H), 3.77 (C*H*N, b, 2H), 3.31 – 2.63 (CH_2N, b, 4H), 2.00 – 1.58 (CCH_2C, b, 4H), 1.45 ($(CH_3)_3$, s, 18H), 1.36 – 0.51 (CCH_2C and CH_3C, m, 29H).

ATR-FT-IR. ν (cm^{-1}): 3316, 3304, 2955, 2926, 2870, 1692, 1518, 1462, 1439, 1389, 1366, 1335, 1308, 1242, 1202, 1167, 1130, 1042, 1020, 991, 953, 928, 903, 858, 756, 727, 665, 627.

HPLC-ESI-MS: *t* = 8.0 – 8.4 minutes, two peaks for multiple isomers, [M+H^+] = 727.2 *m/z*, [M+Na^+] = 749.6 *m/z*, [M-Boc+H^+] = 627.5 *m/z*, [M-tBu+H^+] = 671.1 *m/z*, [M-Boc-tBu+H^+] = 571.4 *m/z*. Calculated: $C_{37}H_{66}N_4O_{10}$ (exact 726.48 g/mol; molar mass 726.95 g/mol).

E. The synthesis of PUI-A1 including analytical data

The synthesis of the asymmetrical poly-urethane **PUI-A1** is similar to that for the previously published **PUI-A2**.

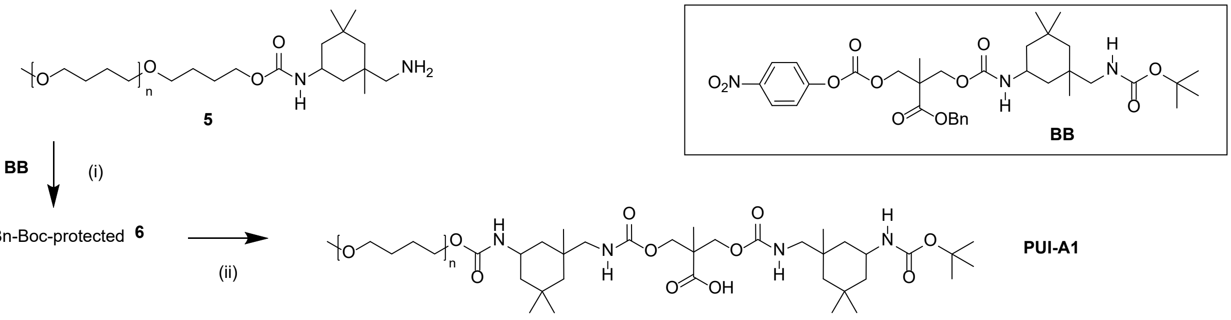

*Scheme S3: Synthesis route to the asymmetric polyether-urethane ionomer **PUI-A1**. (i) **BB**, pyridine, dioxane, reflux; (ii) Pd/C, H_2, dioxane. Note that the IPDA building blocks exist in multiple regio- and stereoisomers; this is not shown for brevity.*

Employing extension building block **BB**, poly-ether mono-amine **5** was converted in 2 steps to **PUI-A1** (Scheme S3). In the coupling reaction, **BB** was used in molar excess. After complete conversion of the coupling reaction, an amine functional scavenger resin was added to the reaction mixture to remove **BB**, and thus separate it from the desired polyurethane product. Debenzylation was required to arrive at the **PUI-A1** end product.

Note that the stepwise syntheses of the PUIs in this paper rely on the use of building block **BB**. It contains (i) a 4-nitrophenyl-carbonate group that is stable at room temperature and that reliable reacts with amines to produce urethane linked products, (ii) a stable benzyl-ester group that can conveniently be deprotected by mild Pd/C-H_2 reduction to give the ionomeric COOH-group, and (iii) a stable Boc-group that allows mild deprotection with TFA to produce a mono-amine reactive group that is suited and ready for the next iterative extension reaction with **BB**.

MeO-polyTHF2000-IPDA-DMPA(Bn)-IPDA-Boc (polymer 6)

Building block molecule **BB** (0.31 g, 0.45 mmol, 2 molar equivalents) was dissolved in dioxane (3 mL) together with MeO-polyTHF2000-IPDA-NH_2 (polymer **5**, 0.7 g, 0.23 mmol) and pyridine (72 µL, 4 equivalents). The mixture was heated to reflux for 16 hours and was kept under an atmosphere of argon. When the reaction was complete (^{1}H-NMR monitoring) a silica gel

functionalized with amine groups (Silicycle Si-Amine catnr. R5203B loading 1.89 mmol/g) was added to react with the excess of building block **BB**. The mixture was stirred for 4 hours at the elevated temperature. After filtration of the reaction mixture to remove the resin, the filtrate was concentrated by evaporation of the solvent. The crude product was dissolved in chloroform, and the organic layer was washed two times with a 0.1M NaOH solution and once with a saturated NaCl solution. The chloroform layer was dried with Na_2SO_4 and concentrated by evaporation of the solvent to yield a yellowish oil (780 mg).

^{1}H NMR (400 MHz, chloroform-d) δ 7.34 (Ar-*H*, m, 5H), 5.17 (CH_2Bn, s, 2H), 4.87 – 4.30 (N*H*, m, 4H), 4.22 (CH_2OCON, d, J = 8.0 Hz, 4H), 4.06 (CH_2CH_2OCON, dd, J = 11.9, 6.1 Hz, 2H), 3.77 (C*H*N, m, 2H), 3.62 – 3.35 (CH_2O, m, ca. 130H), 3.33 (CH_3O, s, 3H), 3.10 – 2.87 (CH_2N, m, 4H), 1.62 (CCH_2C and CH_2CH_2O, m, ca. 140H), 1.44 (($CH_3)_3$, s, 9H), 1.40 – 0.70 (CCH_2C and CH_3C, m, 29H).

SEC (THF-RI): M_n= 6.9 kD, M_w = 8.3 kD, PD = M_w/M_n = 1.21.

Found MALDI-TOF-MS array (CHCA matrix, positive linear mode, broad peaks): (M + Na^+) = ... 2312.1, 2384.3, 2456.5 ... (p = 20-22) for 847.10 + [72.11]p + 22.99 and minor array (M + K^+) = ... 2330.0, 2401.9, 2473.4 ... (p = 20-22) for 847.10 + [72.11]p + 39.09. Calculated molecular weight of end group ($C_{45}H_{74}N_4O_{11}$; n=1): 847.10. Exact: 846.54.

MeO-polyTHF2000-IPDA-DMPA-IPDA-Boc ("PUI-A1")

MeO-pTHF2000-IPDA-DMPA(Bn)-IPDA-Boc (polymer **6**, 630 mg) and Pd/C Degussa type catalyst (100 mg) were mixed in dioxane (4 mL). The reaction mixture was stirred overnight at room temperature under a hydrogen atmosphere. When the conversion was complete (check with ^{1}H-NMR for removal of the benzyl ester) the reaction mixture was filtrated over a zeolite plug to remove the Pd-catalyst. After evaporation of the solvent the product was obtain as a yellowish clear oil (491 mg; 80%). The lower yield was due to the presence of remaining solvent in the starting polymer **6**.

^{1}H NMR (400 MHz, chloroform-d) δ 5.11 – 4.43 (COO*H* and N*H*, m, 5H), 4.22 (CH_2OCON, s, 4H), 4.07 (CH_2CH_2OCON, m, 2H), 3.75 (C*H*N, m, 2H), 3.55 – 3.35 (CH_2O, m, ca. 135H), 3.33 (CH_3O, s, 3H), 2.90 (CH_2N, m, 4H), 1.62 (CCH_2C and CH_2CH_2O, m, ca. 145H), 1.45 (($CH_3)_3$, s, 9H), 1.38 – 0.72 (CCH_2C and CH_3C, m, 29H).

ATR-FT-IR: ν (cm^{-1}): ca. 3300, 2941, 2860, 1711, 1524, 1456, 1371, 1304, 1238, 1211, 1169, 1107, 1047, 1011, 995, 961, 901, 866, 831, 810, 772, 746, 727, 696, 667, 640.

SEC (THF-RI): M_n= 7.4 kD, M_w = 8.6 kD, PD = M_w/M_n = 1.16.

Found MALDI-TOF-MS array (CHCA matrix with added KAc, positive linear mode): $(M + K^{+})$ = ... 2094.3, 2165.6, 2238.5, 2310.8 ... (p = 18-21) for 756.98 + [72.11]p + 39.09. Calculated molecular weight of end group ($C_{38}H_{68}N_4O_{11}$; n=1): 756.98. Exact: 756.49.

F. Compiled analytical data on PU-S0, PUI-S2 and PUI-A2

Boc-IPDA-pTHF2000-IPDA-Boc ("PU-S0")

^{1}H NMR (400 MHz, chloroform-*d*) δ 4.57 (N*H*, d, 4H), 4.05 (CH_2OCON, t, *J* = 6.2 Hz, 4H), 3.75 (C*H*N, m, 2H), 3.65 – 3.20 (CH_2O, m, ca. 100H), 2.86 (CH_2N, d, *J* = 6.7 Hz, 4H), 1.95 – 1.53 (CCH_2C and CH_2CH_2O, m, ca. 105H), 1.44 ((CH_3)$_3$, s, 18H), 1.36 – 0.72 (CCH_2C and CH_3C, m, 26H).

ATR-FT-IR. ν (cm^{-1}): 3327, 2940, 2855, 1717, 1520, 1456, 1439, 1366, 1240, 1168, 1105, 1045, 1013, 995, 959, 907, 860, 773, 750, 667, 654.

SEC (THF-RI): M_n= 5.5 kD, M_w = 8.6 kD, PD = 1.57.

Found MALDI-TOF-MS array (CHCA matrix with added KAc, positive linear mode): (M + Na^+) = ... 1569.7, 1642.1, 1714.0, 1786.0 ... (p = 12-15) for 682.94 + [72.11]p + 22.99 and minor array (M + K^+) = ... 1586.4, 1658.9, 1730.6, 1802.8 ... (p = 12-15) for 682.94 + [72.11]p + 39.09. Calculated molecular weight of end group ($C_{36}H_{66}N_4O_8$; n=0): 682.94. Exact: 682.49.

MeO-polyTHF2000-IPDA-DMPA-IPDA-DMPA-IPDA-Boc ("PUI-A2")

^{1}H NMR (400 MHz, chloroform-*d*) δ 5.4 – 4.4 (COO*H* and N*H*, m, 8H), 4.22 (CH_2OCON, m, 8H), 4.06 (CH_2CH_2OCON, m, 2H), 3.75 (C*H*N, m, 3H), 3.60 – 3.35 (CH_2O, m, ca. 130H), 3.33 (CH_3O, s, 3H), 3.10 – 2.85 (CH_2N, m, 6H), 1.95 – 1.50 (CCH_2C and CH_2CH_2O, m, ca. 140H), 1.44 ((CH_3)$_3$, s, 9H), 1.35 – 0.70 (CCH_2C and CH_3C, m, 45H).

ATR-FT-IR. ν (cm^{-1}): ca. 3300, 2938, 2853, 1721, 1713, 1530, 1456, 1439, 1366, 1343, 1306, 1238, 1105, 1045, 984, 959, 756, 733, 702, 633, 615.

SEC (THF-RI): M_n= 7.2 kD, M_w = 8.5 kD, PD = 1.19.

Found MALDI-TOF-MS array (CHCA matrix with added KAc, positive linear mode): (M + K^+) = ... 2017.2, 2090.5, 2161.5, 2234.5 ... (p = 12-15) for 1113.40 + [72.11]p + 39.09. Calculated molecular weight of end group ($C_{55}H_{96}N_6O_{17}$; n=1): 1113.40. Exact: 1112.68.

Boc-IPDA-DMPA-IPDA-pTHF2000-IPDA-DMPA-IPDA-Boc ("PUI-S2")

^{1}H NMR (400 MHz, chloroform-*d*) δ 5.15 – 4.50 (COO*H* and N*H* , br, 10H), 4.3 – 4.1 (CH_2OCON , m, 8H), 4.1 – 4.0 (CH_2OCON, m, 4H), 3.85 – 3.60 (C*H*N, 4H), 3.41 (CH_2O, m, ca. 105H), 2.9 – 2.7 (CH_2N , m, 8H), 1.75 – 1.50 (CCH_2C and CH_2CH_2O, m, ca. 115H), 1.45 ((CH_3)$_3$, s, 18H), 1.3 – 0.75 (CCH_2C and CH_3, m, 58H).

ATR-FT-IR. ν (cm^{-1}): ν (cm^{-1}): 3314, 2940, 2855, 1713, 1530, 1458, 1439, 1366, 1306, 1236, 1169, 1107, 1044, 986, 959, 754, 696, 667, 646, 633.

SEC (THF-RI): M_n= 6.4 kD, M_w = 8.8 kD, PD = 1.37.

Found MALDI-TOF-MS array (CHCA matrix with added KAc, positive linear mode): ($M + K^+$) = ... 1795.1, 1867.6, 1939.4, 2011.6 ... (p = 5-8) for 1395.78 + [72.11]p + 39.09. Calculated molecular weight of end group ($C_{70}H_{122}N_8O_{20}$; n=1): 1395.78. Exact: 1394.88.

G. Analytical data on the sequence-defined PU(I)s: MS, SEC and NMR figures and tables

(i) HPLC-MS and MALDI-TOF-MS data

(ii) SEC data

(iii) NMR information

(i) HPLC-MS and MALDI-TOF-MS data

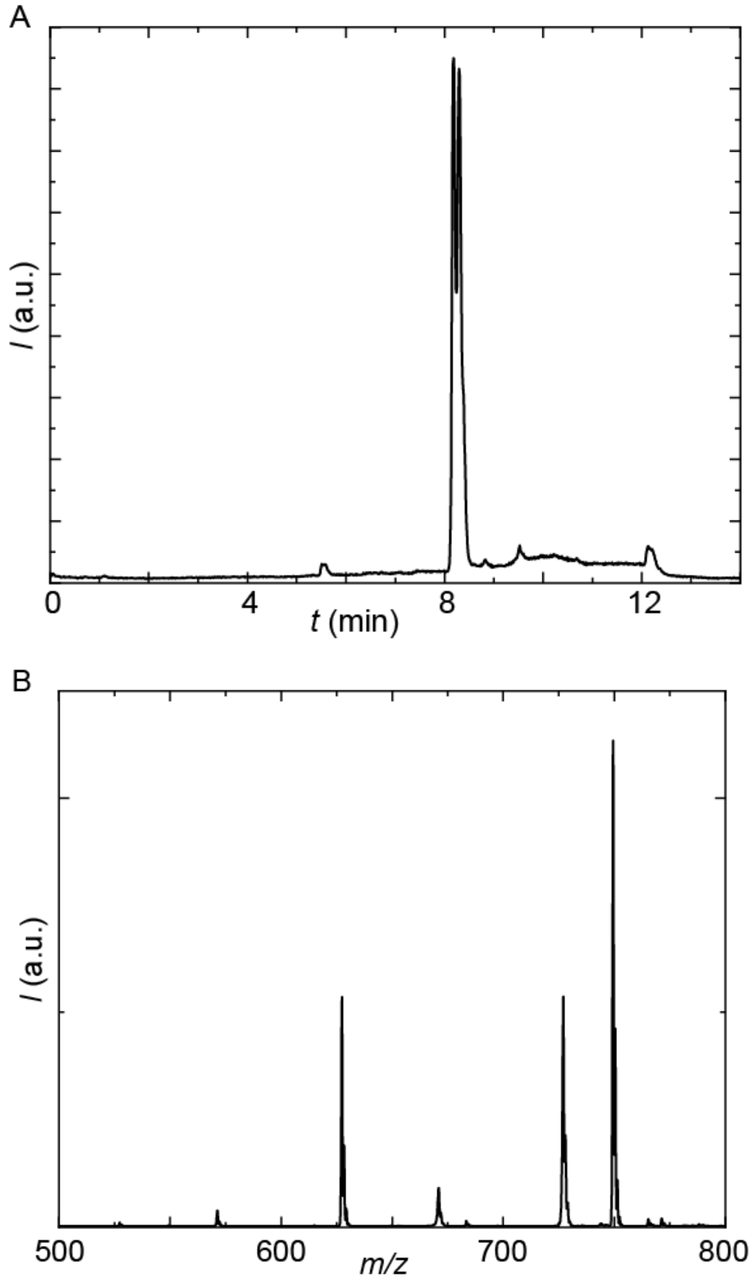

*Figure S1: HPLC-MS chromatogram and MS spectrum of **sPUI-S1**. A) Chromatogram with two peaks at about t = 8.2 minutes due to **sPUI-S1** being an isomeric mixture. B) MS-spectrum (t = 8.0 to 8.4 minutes), m/z [M+H$^+$] = 727.2, [M+Na$^+$] = 749.6, [M-Boc+H$^+$] = 627.5, [M-tBu+H$^+$] = 671.1, [M-Boc-tBu+H$^+$] = 571.4.*

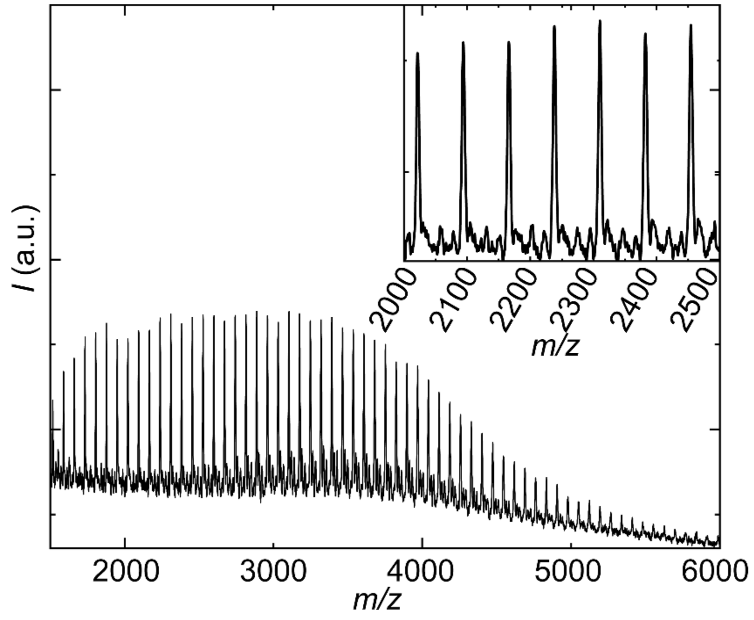

*Figure S2: MALDI-TOF-MS of **PUI-A1.** The inset is a zoom-in of the MS-spectrum: (M + K$^+$) = ... 2094.3, 2165.6, 2238.5, 2310.8 ... (p = 18-21) for 756.98 + [72.11]p + 39.09. Matrix: CHCA with added KAc. Mode: positive linear.*

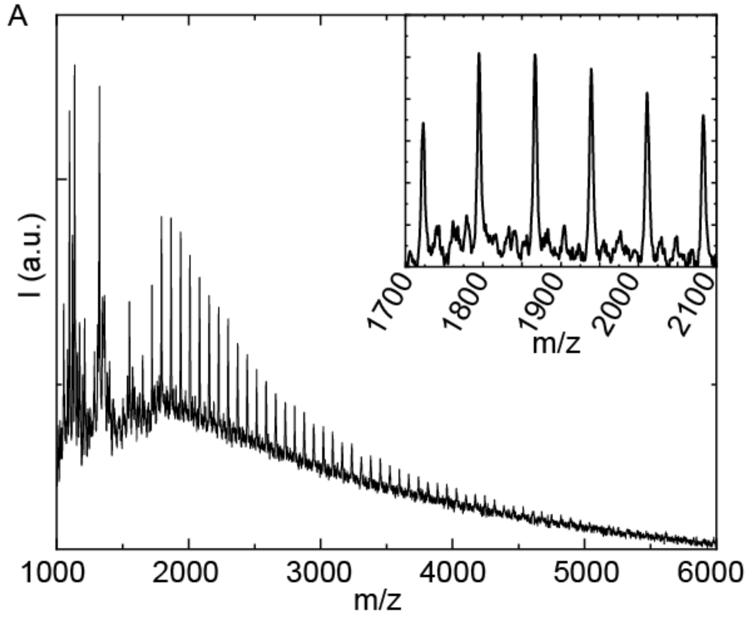

*Figure S3: MALDI-TOF-MS of **PUI-S2**. Matrix: CHCA with added KAc. Mode: positive linear. The inset is a zoom-in of the MS-spectrum: (M + K+) = ... 1795.1, 1867.6, 1939.4, 2011.6 ... (p = 5-8) for 1395.78 + [72.11]p + 39.09.*

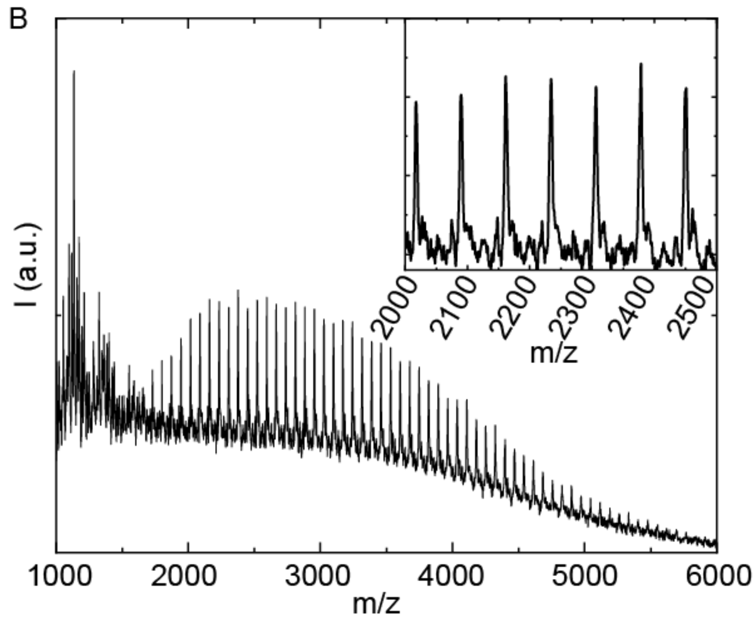

Figure S4: MALDI-TOF-MS of ***PUI-A2****. Matrix: CHCA with added KAc. Mode: positive linear. The inset is a zoom-in of the MS-spectrum: (M + K^+) = ... 2017.2, 2090.5, 2161.5, 2234.5 ... (p = 12-15) for 1113.40 + [72.11]p + 39.09.*

(ii) SEC data

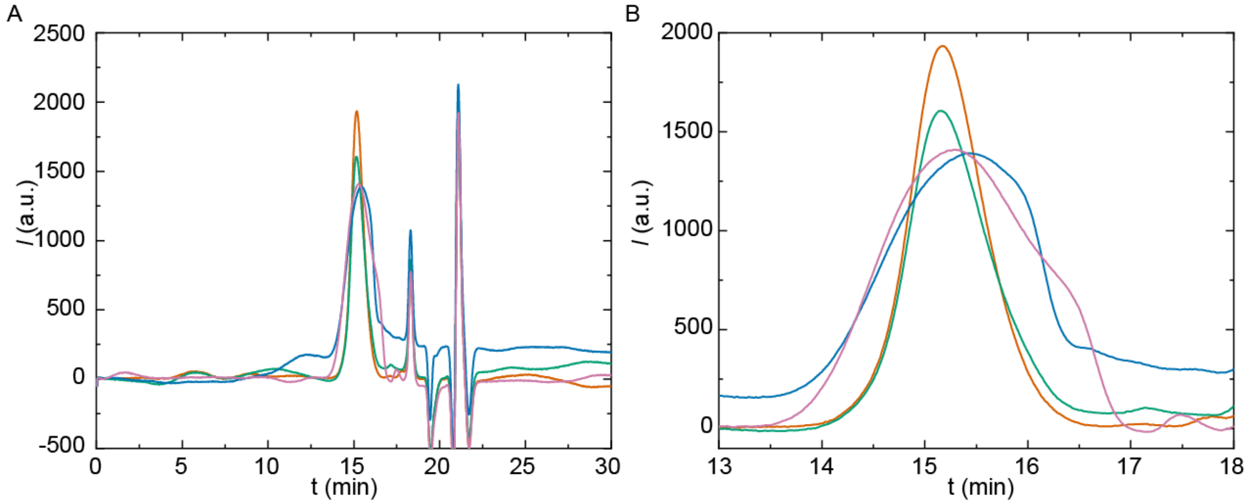

Figure S5: SEC traces of ***PU-S0*** *(pink),* ***PUI-A1*** *(orange),* ***PUI-A2*** *(green) and* ***PUI-S2*** *(blue). A) Full trace. B) Zoom-in of product peaks. Eluent: THF. Detection: refractive index.*

Table S1: Compilation SEC data for ***PU-S0***, ***PUI-A1***, ***PUI-A2*** *and* ***PUI-S2***. *The molecular weight and distribution data are relative to polystyrene (PS) standards. Eluent: THF. Detection: RI.*

**Material**	**M_n** (kDa)	**M_w** (kDalton)	**PD or M_w/M_n** (-)
**PU-S0**	5.46	8.57	1.57
**PUI-A1**	7.38	8.58	1.16
**PUI-A2**	7.16	8.53	1.19
**PUI-S2**	6.40	8.76	1.37

Figure S4 and Table S1 show that the asymmetric **PUI-A1** and **PUI-A2** are more narrowly distributed than the symmetric **PU-S0** and **PUI-S2**. Apparently, the commercially available dihydroxy telechelic poly-tetrahydrofuran (M_W = 2000), used as the starting point to prepare the symmetric PU(I)s, has a broader distribution than the cationically polymerized mono-hydroxy poly-tetrahydrofuran (M_W = 2000) that was prepared as precursor to the asymmetric PUIs (see reference 1). Furthermore, the asymmetric PUIs have a similar hydrodynamic volume in THF eluent, even though **PUI-A2** has a higher molecular weight. This may be due to the increased possibilities for intramolecular hydrogen bonding within **PUI-A2** in THF.

(iii) NMR information

Due to the presence of the IPDA units in the prepared PU(I)s, the ^{1}H-NMR data become complicated as various isomers give resonances at different positions. The below gives an overview of the proton assignments, where the given virtual molecule is a simplified structure that represents the variety of protons that are typically found in the prepared set of PU(I)s.

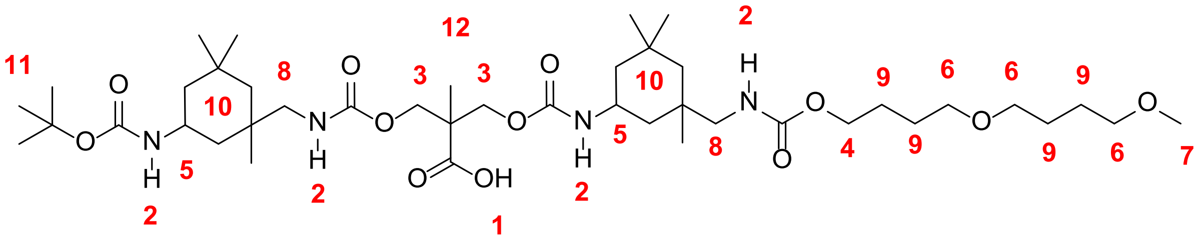

Table S2.: ^{1}H NMR assignment

Chemical shift (ppm)	Position	Type of proton	Notes	# Protons (per unit)	Unit
6.0 – 4.6	1, 2	COO*H* and N*H*	multiple signals, br	1H and 2H	DMPA and IPDA
4.22	3	CH_2OCON	s, or multiple signals	4H	DMPA
4.05	4	CH_2CH_2OCON	t, or multiple signals	2H	THF-monomer
3.85 – 3.70	5	*C*HN	multiple signals	1H	IPDA
3.55 – 3.35	6	CH_2O	multiple signals	4H	THF-monomer
3.33	7	CH_3O	s	3H	MeO end group
3.0 – 2.6	8	CH_2N	multiple signals	2H	IPDA
1.6	10	CCH_2C	multiple signals	2H	IPDA
1.8 – 1.5	9	CH_2CH_2O	multiple signals	4H	THF-monomer
1.44	11	C$(CH_3)_3$	s	9H	Boc
1.1 – 1.0	12	CH_3C	s, or multiple s	3H	DMPA
1.3 – 0.65	10	CCH_2C and CH_3C	multiple signals	13H	IPDA

Table S3: Aliphatic region assignment details for IPDA and DMPA units

Chemical shift (ppm)	Type of proton (notes)	Unit (protons per unit)
1.6 / 1.25 / 1.05 / 0.90	CCH_2C (multiple signals)	IPDA (6H)
1.1 – 1.0	CH_3 (s or multiple s)	DMPA (3H)
1.3 – 0.65	CH_3C (multiple s)	IPDA (9H)

^{1}H-NMR can be used the calculate and estimate the average number molecular weight M_n of the poly-THF segment within the PU(I) sequences, and therefore also the M_n of the PU-material (Table S4 and Table S5). The actual ^{1}H-NMR spectra of the PUs are compiled in ESI Section 7.

Briefly, the integral in the 1.5-0.6 ppm region is used to calculate the integral per proton. Next, the integrals of the signals at about 3.4 and 1.6 ppm (of the pTHF units) are used to calculate the average number of pTHF units in the PU-sequence. Note that the integral of the pTHF units is corrected as the signal at 3.4 ppm also accounts for the 3 methoxy-protons (in **PUI-A1** and **PUI-A2**) and does not account for the C*H*$_2$-OCONH protons (2 or 4 for **PUI-A1** and **PUI-A2** or **PU-S0** and **PUI-S2**, respectively), while the signal at 1.6 ppm also accounts for 2 protons of every IPDA unit.

Table S4.: ^{1}H NMR assessment of the number of poly-THF units within the prepared PUs

Material	Integral 1.5-0.6 ppm	Representing # protons	Integral per 1H	Integral pTHF	Integral corrected pTHF	pTHF # units
**PU-S0**	42.27	44	0.961	207.92	207.92	27.1
**PUI-S2**	71.23	76	0.937	202.51	198.76	26.5
**PUI-A1**	35.6	38	0.937	249.46	242.90	32.4
**PUI-A2**	54.44	54	1.008	282.38	271.44	33.7

Table S5.: Molecular weights (M_n) of poly-THF and of PU-materials as calculated by ^{1}H NMR

Material	pTHF # units	pTHF Mn (Dalton)	PU-material Mn (Dalton)
**PU-S0**	27.1	1951	2562
**PUI-S2**	26.5	1911	3235
**PUI-A1**	32.4	2337	3022
**PUI-A2**	33.7	2427	3468

3. SCF information

Table S6. Overview of Flory-Huggins χ-parameters used in the SCF computations, following Li et al. (reference 4)

	H_2O	C	O	C_3	COO^-	NH	C=O	Na (=Cl)
H_2O	0	1.6	-0.7	2.3	0	1.6	-0.7	0
C		0	1	0.6	1.6	0	-1	1.6
O			0	1.6	-0.7	1	1.6	-0.7
C_3				0	2.3	0.6	-1	2.3
COO^-					0	1.6	2.3	0
NH						0	-1.6	1.6
C=O							0	1.6
Na (=Cl)								0

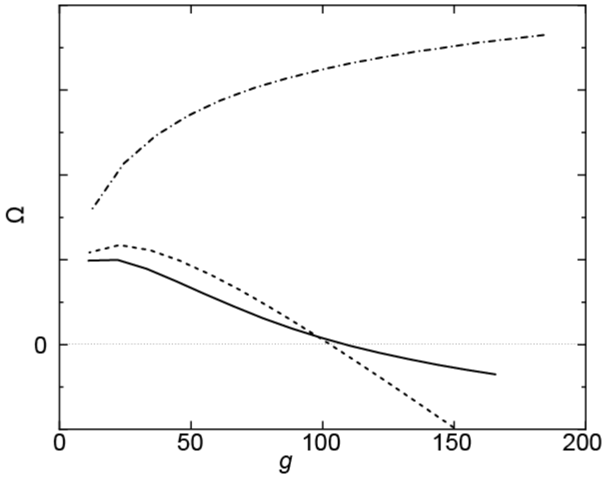

*Figure S6: Grand potential Ω as a function of aggregation number g, showing how **PUI-A2** (dotted) and **PUI-S2** (solid) cross zero (indicative of solubilization by micelle formation), whereas **PUI-A1** (dash dot) does not (indicative of insolubility in water).*

4. Cryo-TEM analyses

Sequence-defined PUs in aqueous 0.1 M TEA solutions were analyzed by cryo-TEM (cryogenic transmission electron microscopy). Dimensions of observed micelle particles were assessed by applying ImageJ software, an open source image processing program, by checking every particle twice, first along the longest axis of the particle and second perpendicular to this axis. Acquired data are collected and shown in Table S7 and Figure S7 to Figure S10. The micelles formed by the combined hosts **PUI-A1** and **sPUI-S1** and the **PU-S0** guest show a broader distribution in radius than the other analyzed micelles.

Table S7. Micelle radius and radius standard deviation information acquired by cryo-TEM on PU solutions in aqueous 0.1 M TEA.

**Sample solution of** [1]	**Cryo-TEM** [2]		
	Radius (nm)	Radius-SD (nm)	Probed micelles (#)
**PUI-A2**	7.2	0.84	19
**PUI-S2**	5.2	1.15	11
**PUI-A1 + sPUI-S1** [3]	4.0	0.77	88
**(PUI-A1 + sPUI-S1) host + PU-S0 guest** [3,4]	7.1	2.9	23

*1. (Cumulative) host concentration is about 2.5 mg/mL in 0.1 M TEA in water; 2. From ImageJ assessements on three independent cryo-TEM pictures; 3. Molar ratio is 1 to 6 for **PUI-A1** to **sPUI-S1**; 4. f_{guest} = 0.195.*

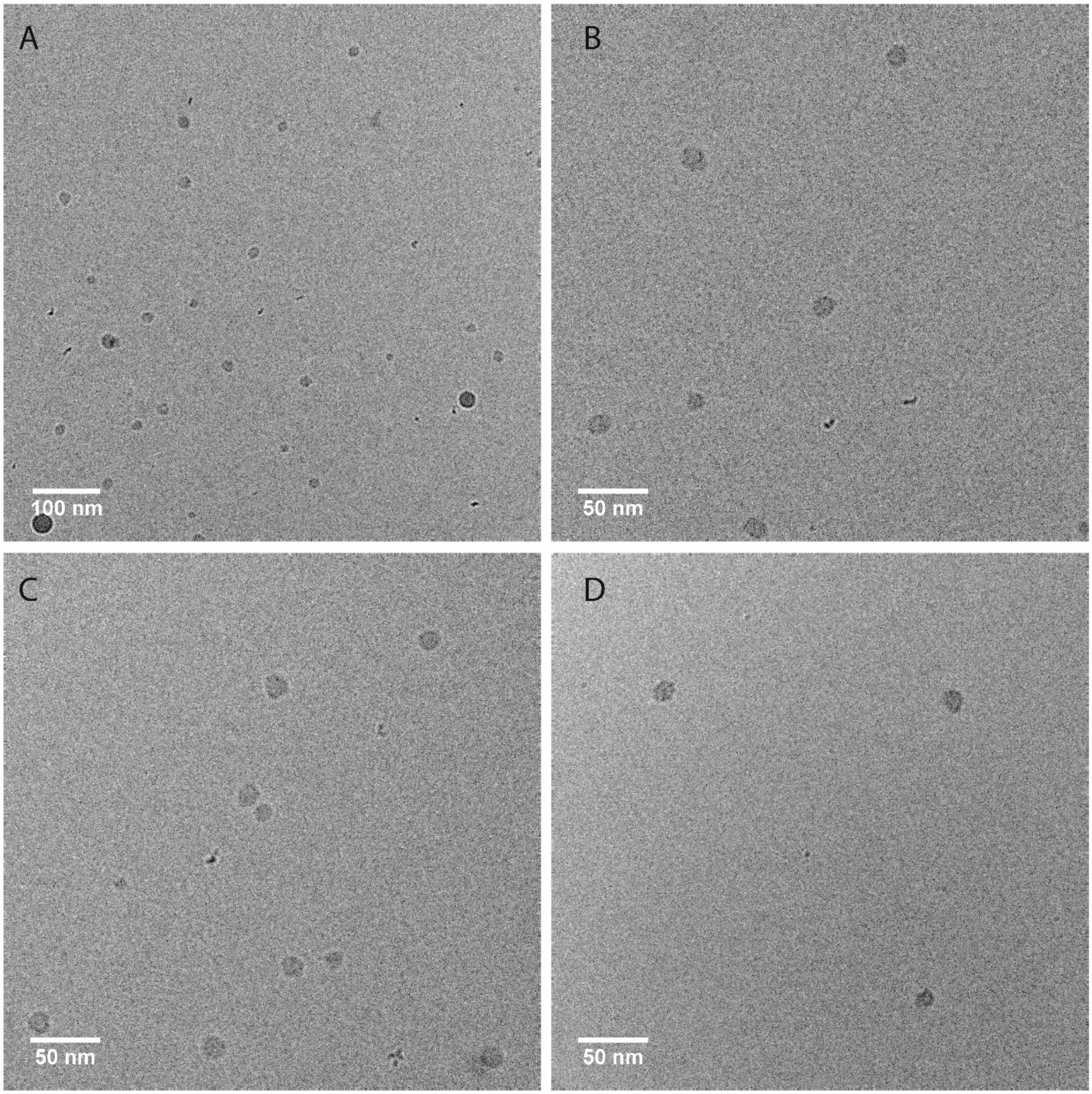

*Figure S7: Cryo-TEM micrographs of **PUI-A2** in aqueous 0.1M TEA solution. The grey spots are vitrified particles in the ice layer, darker spots are ice particles at the surface. Concentration: approximately 2.5 mg/mL. A. Magnification: 24000. B. Same picture as A, magnification: 48000. C. Other picture as A, magnification: 48000. D. Other picture as A or C, magnification: 48000.*

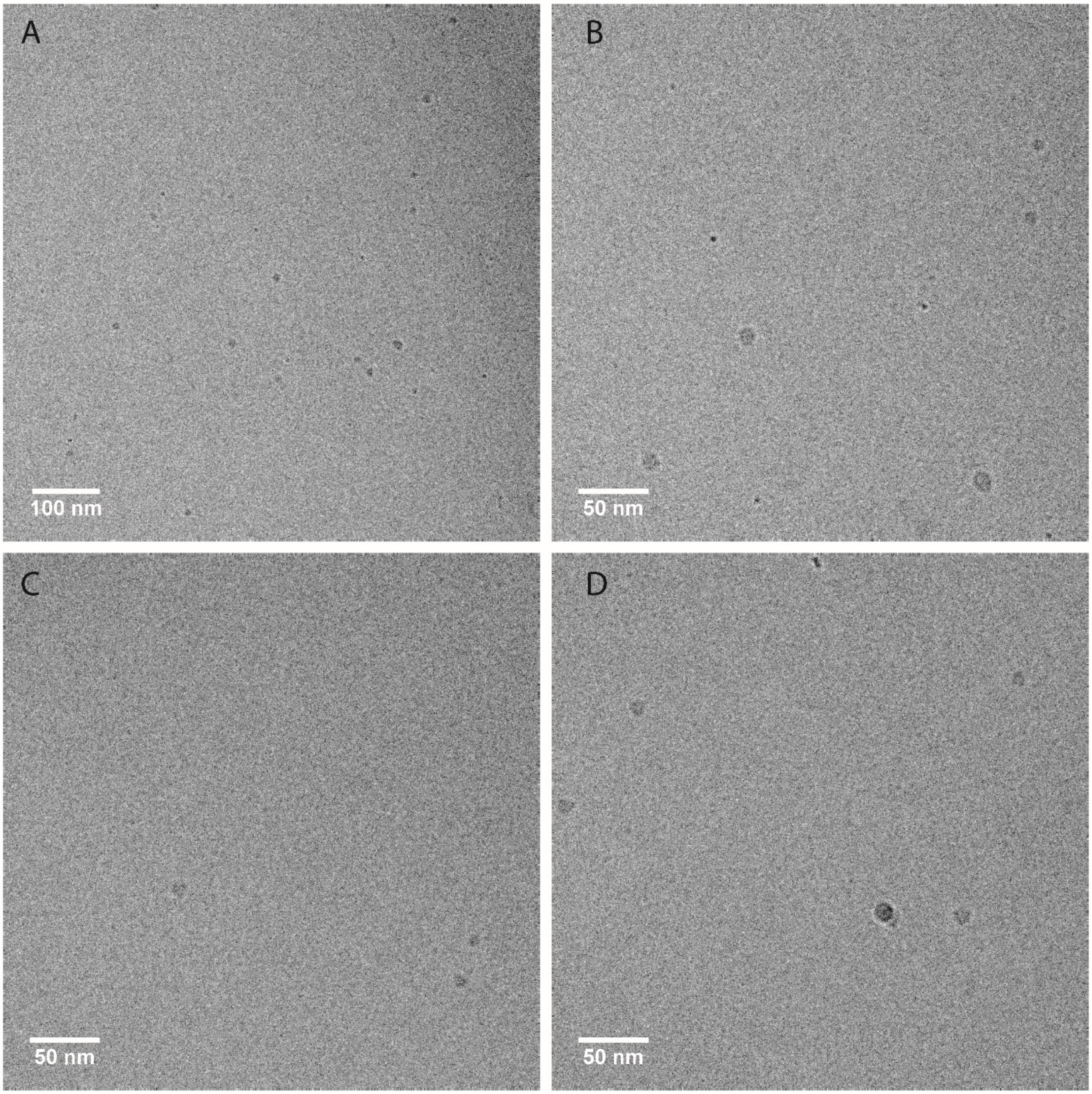

Figure S8: Cryo-TEM micrographs of ***PUI-S2*** *in aqueous 0.1M TEA solution. The grey spots are vitrified particles in the ice layer, darker spots are ice particles at the surface. Concentration: approximately 2.5 mg/mL. A. Magnification: 24000. B. Same picture as A, magnification: 48000. C. Other picture as A, magnification: 48000. D. Other picture as A or C, magnification: 48000.*

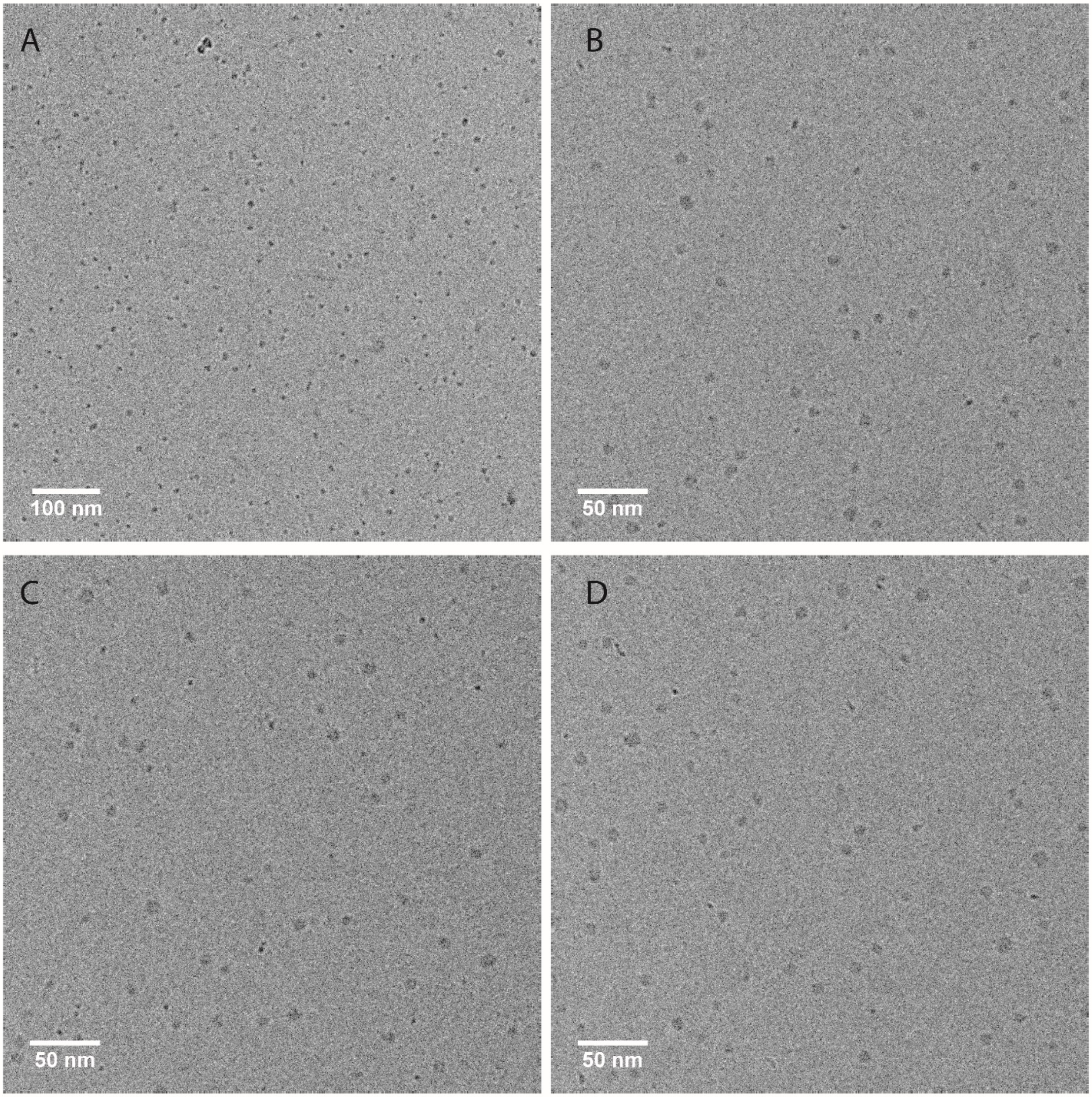

Figure S9: Cryo-TEM micrographs of ***PUI-A1*** *with* ***sPUI-S1*** *in a 1:6 molar ratio in aqueous 0.1M TEA solution. The grey spots are vitrified particles in the ice layer, darker spots are ice particles at the surface. Concentration: approximately 2.5 mg/mL. A. Magnification: 24000. B. Same picture as A, magnification: 48000. C. Other picture as A, magnification: 48000. D. Other picture as A or C, magnification: 48000.*

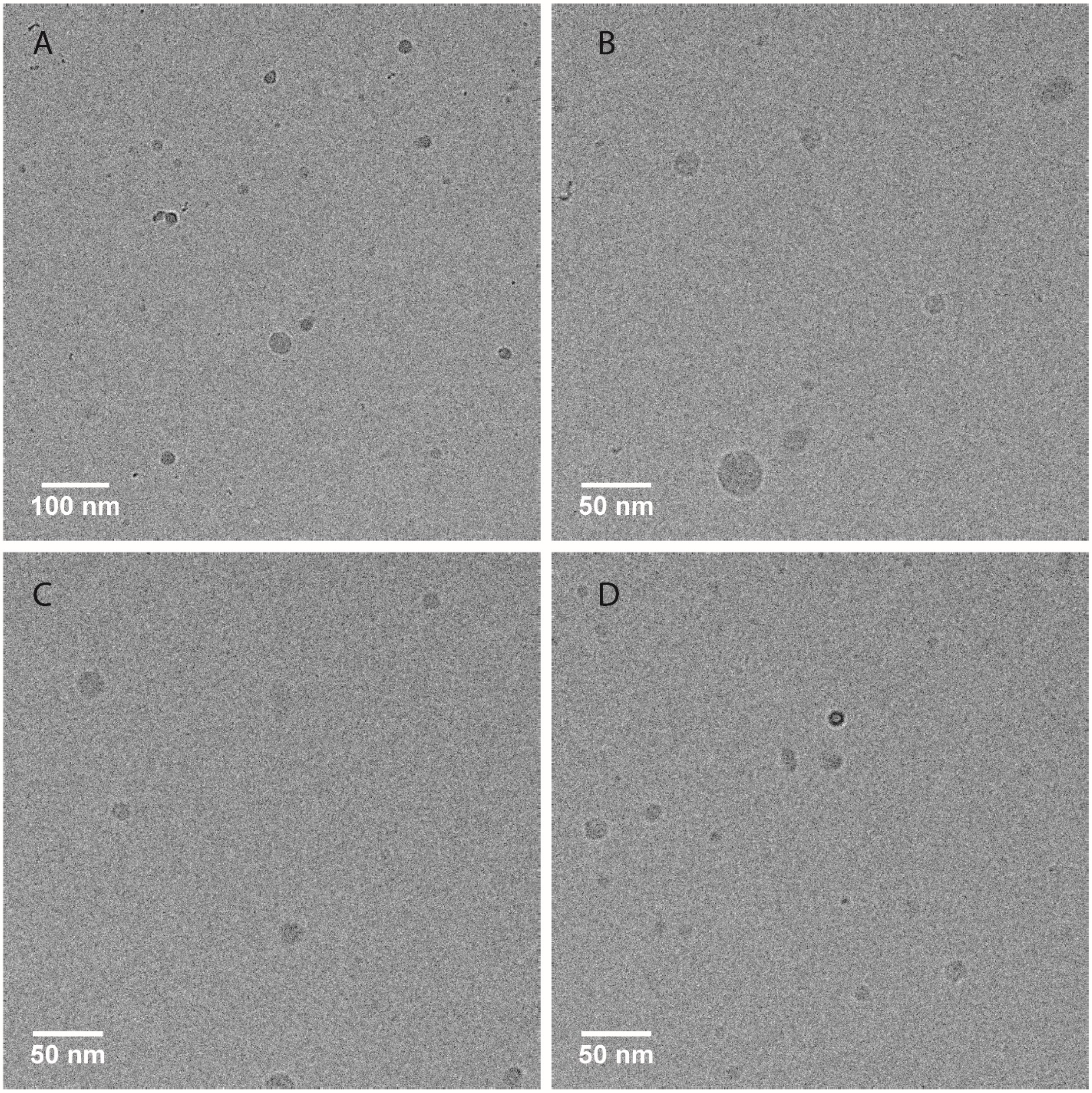

*Figure S10: Cryo-TEM micrographs of **PUI-A1** with **sPUI-S1** in a 1:6 molar ratio as combined host with added **PU-S0** guest (f_{guest} = 0.195) in aqueous 0.1M TEA solution. The grey spots are vitrified particles in the ice layer, darker spots are ice particles at the surface. Cumulative concentration hosts: approximately 2.5 mg/mL. A. Magnification: 24000. B. Same picture as A, magnification: 48000. C. Other picture as A, magnification: 48000. D. Other picture as A or C, magnification: 48000.*

5. DSC information

The PU(I) materials were analyzed using a TA Q2000 DSC machine. Data were thermally analyzed using Universal Analysis software.

Samples were heated at 10 ^{0}C/min in the first heating run to 80 ^{0}C or 100 ^{0}C. Thereafter and subsequently, a cyle at a scanning rate of 10 ^{0}C/min and a cycle at a scanning rate of 40 ^{0}C/min. were recorded. Cooling runs went to -85 ^{0}C. In the below, the thermal results are compiled. The DSC traces are shown in ESI Section 7.

sPUI-S1. White fully amorphous powder. T_g = 58.7 ^{0}C (heating run 40 ^{0}C/min).

PU-S0. White semi-crystalline material. Melt in the first heating run at T_m = 34.9 ^{0}C (33.5 J/g). Partial crystallization in first cooling run. Then recrystallization at T_{cr} = -15.8 ^{0}C (31.5 J/g) and subsequent T_m = 21.9 ^{0}C (58.7 J/g) in the second heating run. These transitions represent melting and (re)crystallization of the poly-THF crystalline phase. Possible T_g of amorphous poly-THF phase at about -65 ^{0}C (heating run 40 ^{0}C/min).

PUI-A1. Thick waxy-oil material. No transition in the first heating run. Partial crystallization in first cooling run at T_{cr} = -11.4 ^{0}C (54.7 J/g). Then minor recrystallization and melt at T_m = 20.8 ^{0}C (67.2 J/g) in the second heating run. These transitions represent melting and (re)crystallization of the poly-THF crystalline phase. No T_g of the amorphous poly-THF phase is observed.

PUI-A2. Thick waxy-oil material. No transition in the first heating run. No crystallization in the first cooling run. Then recrystallization at T_{cr} = -13.3 ^{0}C (35.3 J/g).) and subsequent melt at T_m = 17.0 ^{0}C (42.7 J/g) in the second heating run. These transitions represent melting and (re)crystallization of the poly-THF crystalline phase. No T_g of the amorphous poly-THF phase is observed.

PUI-S2. Thick waxy oil material. No transitions are observed. Fully amorphous material. Apparently, the end groups prevent the poly-THF phase from crystallizing.

6. References

1. Timmers, E.M.; Fransen, P.M.; Magana, J.R.; Janssen, H.M.; Voets, I.K. Micellization of Sequence-Controlled Polyurethane Ionomers in Mixed Aqueous Solvents. *Macromolecules*, **2021**, *54*, 2376–2382. https://dx.doi.org/10.1021/acs.macromol.0c02107.

2. Söntjens, S.H.M.; Renken, R.A.E.; van Gemert, G.M.L.; Engels, T.A.P., Bosman, A.W.; Janssen, H.M.; Govaert, L.E.; Baaijens, F.B.T. Thermoplastic elastomers based on strong and well-defined hydrogen-bonding interactions. *Macromolecules* **2008**, *41*, 15, 5703–5708. https://doi.org/10.1021/ma800744c.

3. Welsh, D.J.; Jones, S. P.; Smith, D. K. "On-Off" Multivalent Recognition: Degradable Dendrons for Temporary High-Affinity DNA Binding. *Angewandte Chemie*, **2009**, *121*, 22, 4107-4111.

4. Li, F.; Tuinier, R.; Van Casteren, I.; Tennebroek, R.; Overbeek, A.; Leermakers, F.A.M. Self-Organization of Polyurethane Pre-Polymers as Studied by Self-Consistent Field Theory. *Macromolecular Theory and Simulations*, **2016**, *25,* 1, 16-27.

7. NMR and IR spectra, DSC thermograms of sPUI-S1, PU-S0, PUI-A1, PUI-A2, PUI-S2

Figure S11: ^{1}H-NMR in $CDCl_3$ of **sPUI-S1**

Figure S12: ^{1}H-NMR in $CDCl_3$ of **PU-S0**

Figure S13: ^{1}H-NMR in $CDCl_3$ of **PUI-A1**

Figure S14: ^{1}H-NMR in $CDCl_3$ of **PUI-A2**

Figure S15: ^{1}H-NMR in $CDCl_3$ of **PUI-S2**

Figure S16: ATR-FT-IR of **sPUI-S1**

Figure S17: ATR-FT-IR of **PU-S0**

Figure S18: ATR-FT-IR of **PUI-A1**

Figure S19: ATR-FT-IR of **PUI-A2**

Figure S20: ATR-FT-IR of **PUI-S2**

Figure S21: DSC thermograms of **sPUI-S1**

Figure S22: DSC thermograms of **PU-S0**

Figure S23: DSC thermograms of **PUI-A1**

Figure S24: DSC thermograms of **PUI-A2**

Figure S25: DSC thermograms of **PUI-S2**

Figures S21-S25: Top picture, first heating run. Bottom picture, 10 ^{0}C/min cycle (lower two traces) and 40 ^{0}C/min heating run (top trace).

sPUI-S1

Figure S11: 1H-NMR

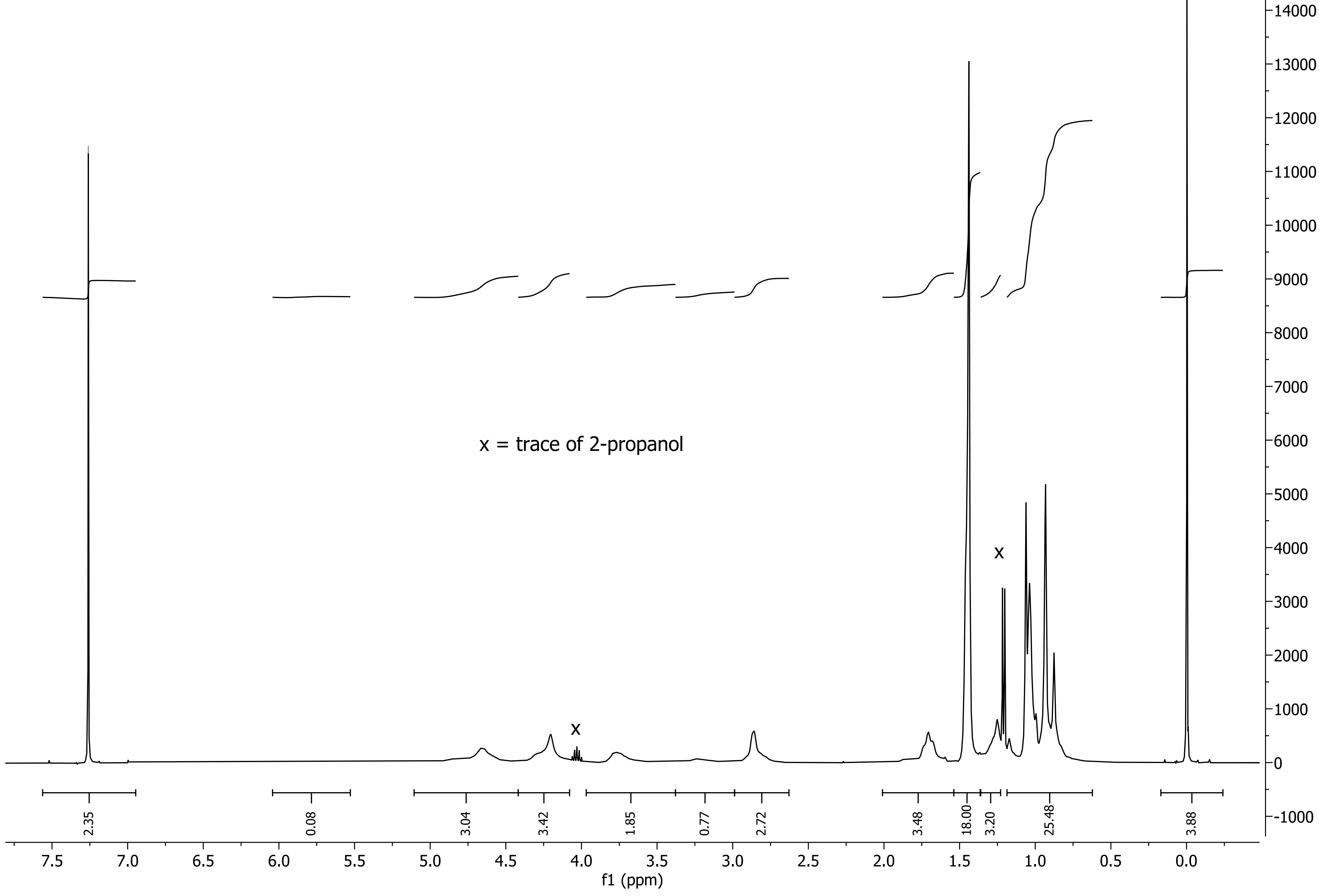

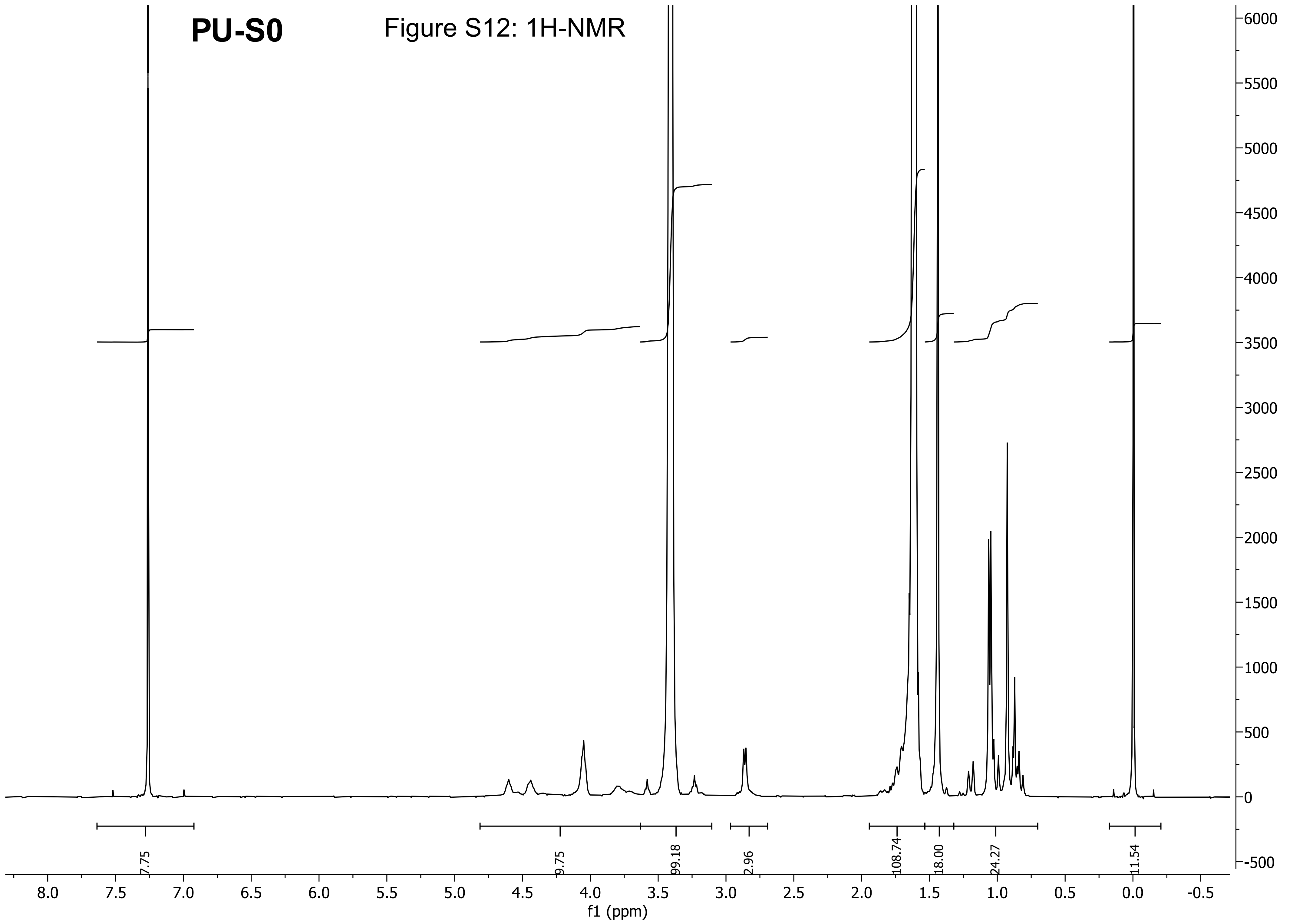

PU-S0
Figure S12: 1H-NMR
7.75
9.75
99.18
2.96
108.74
18.00
24.27
11.54
f1 (ppm)

PUI-A1

Figure S13: 1H-NMR

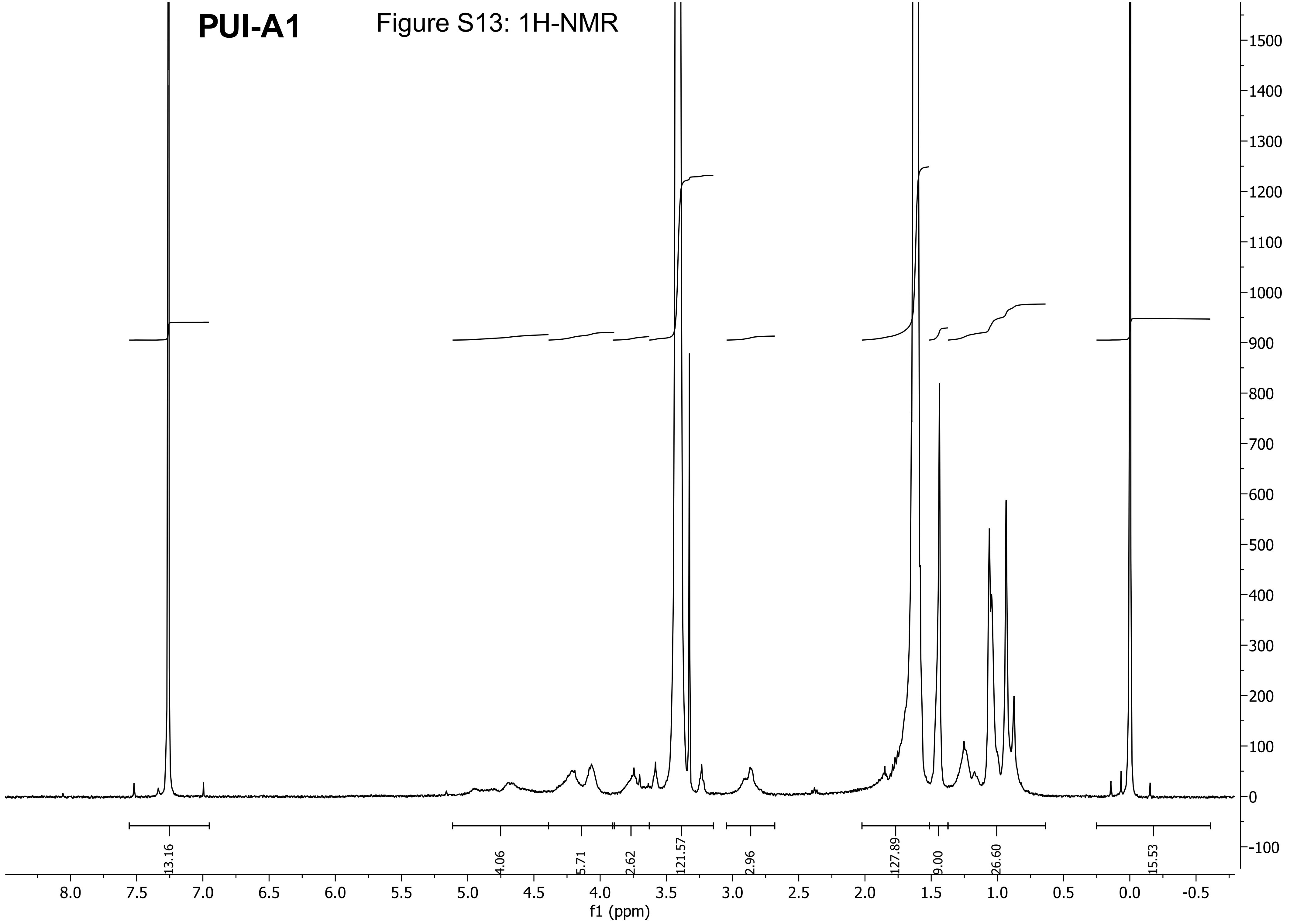

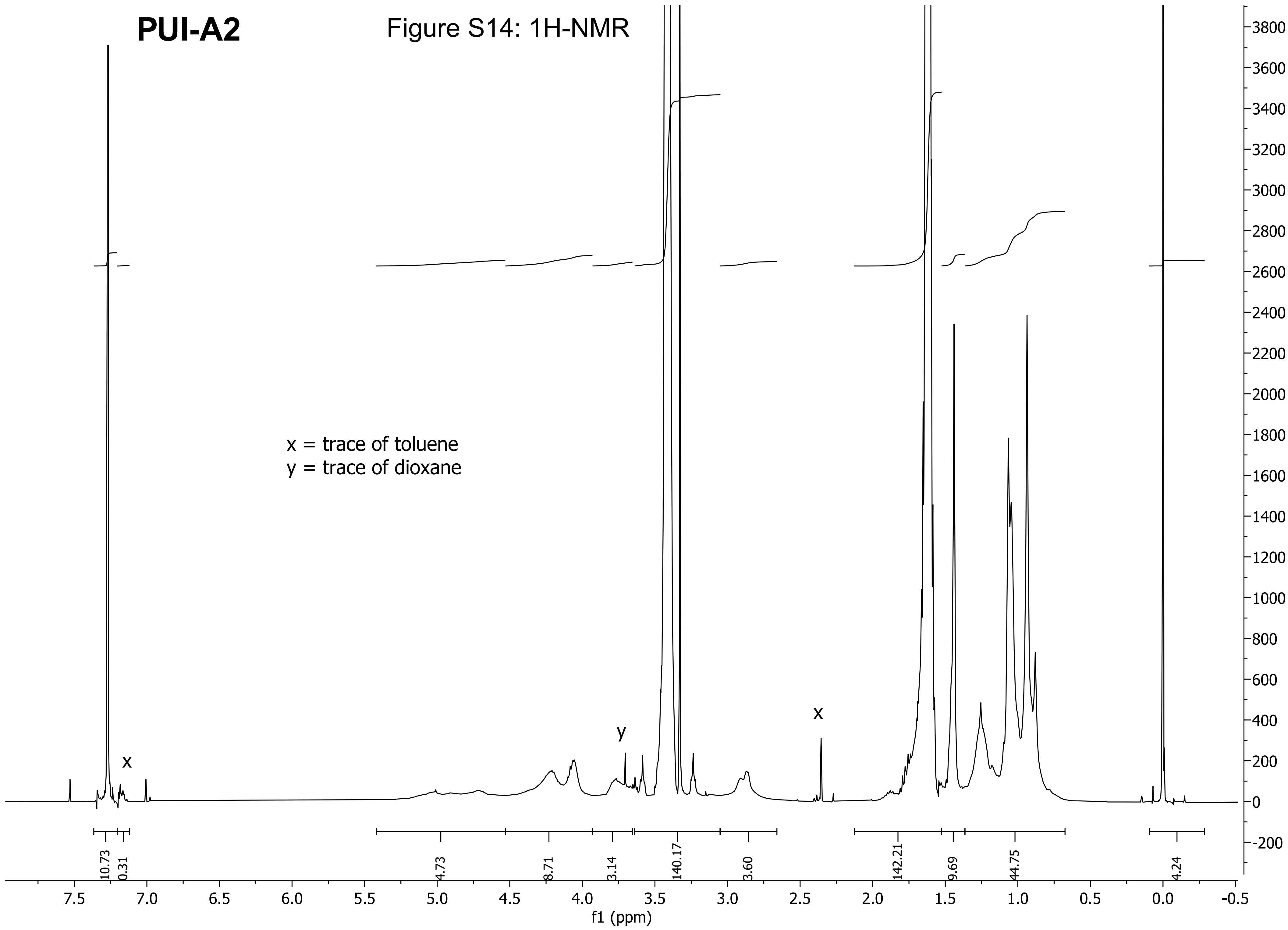
PUI-A2
Figure S14: 1H-NMR
x = trace of toluene
y = trace of dioxane
x
y
x
10.73
0.31
4.73
8.71
3.14
140.17
3.60
142.21
9.69
44.75
4.24
f1 (ppm)

PUI-S2

Figure S15: 1H-NMR

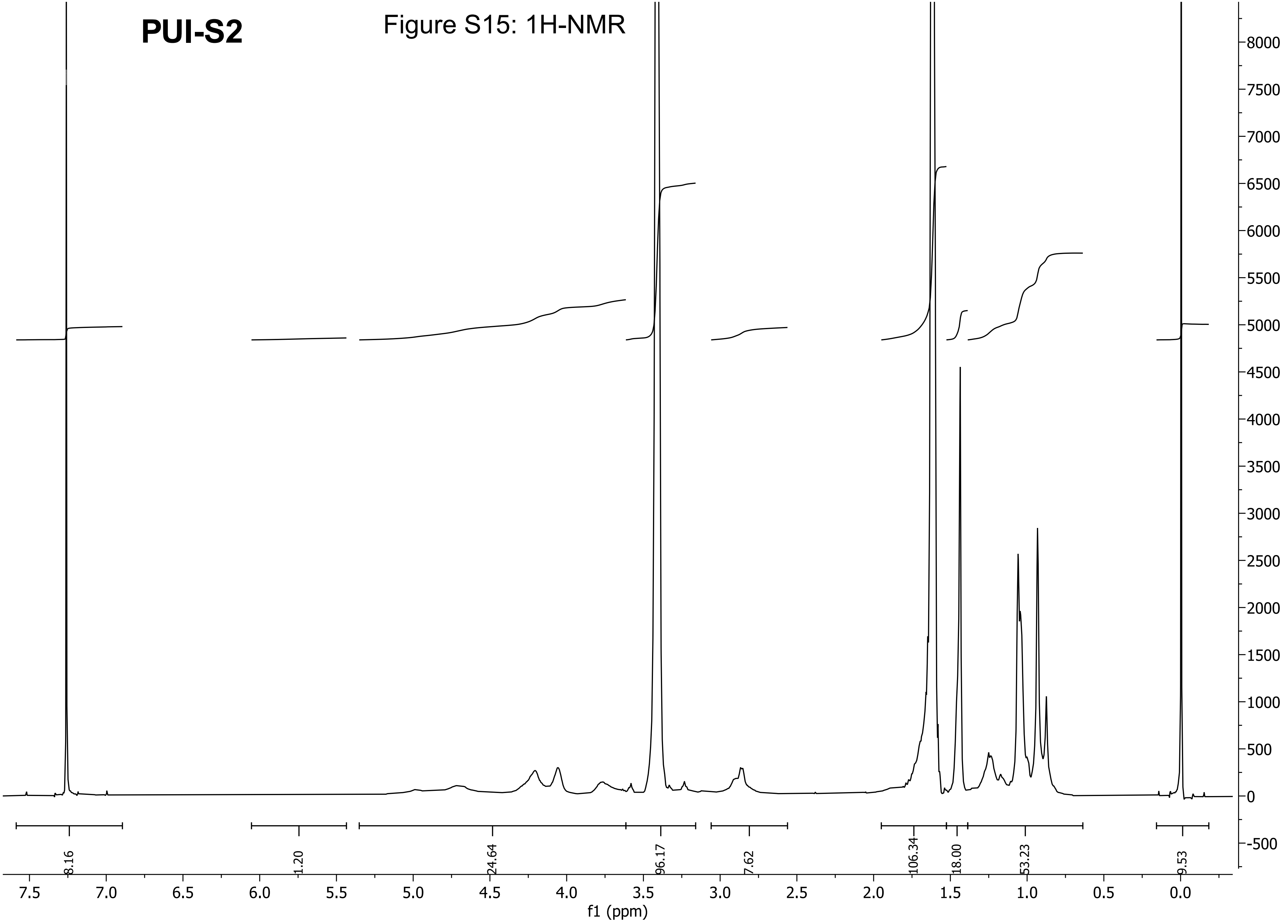

sPUI-S1

Figure S16: ATR-FT-IR

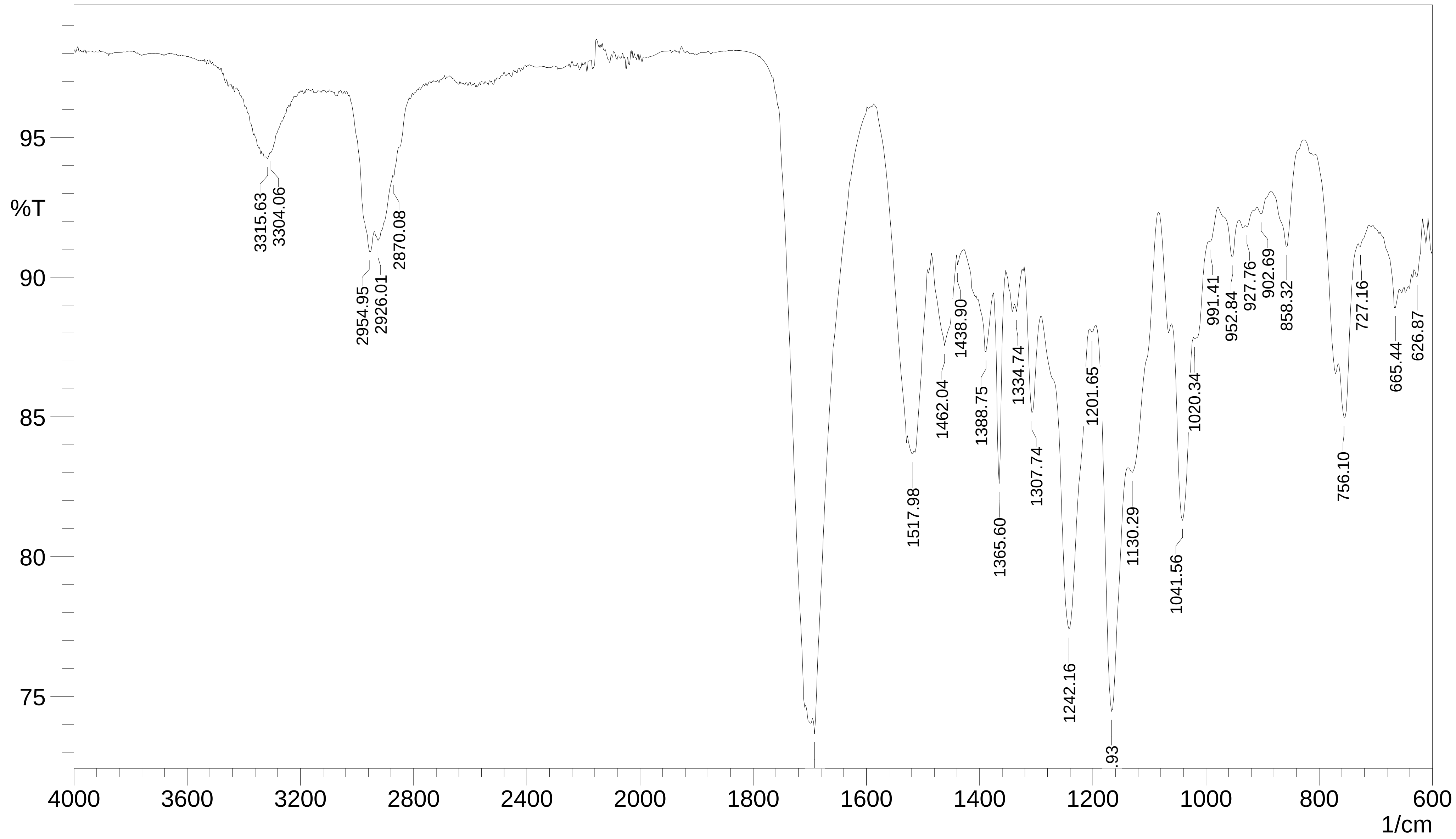

No. of Scans; 20

Resolution; 4 [1/cm]

Apodization; Happ-Genzel

PU-S0

Figure S17: ATR-FT-IR

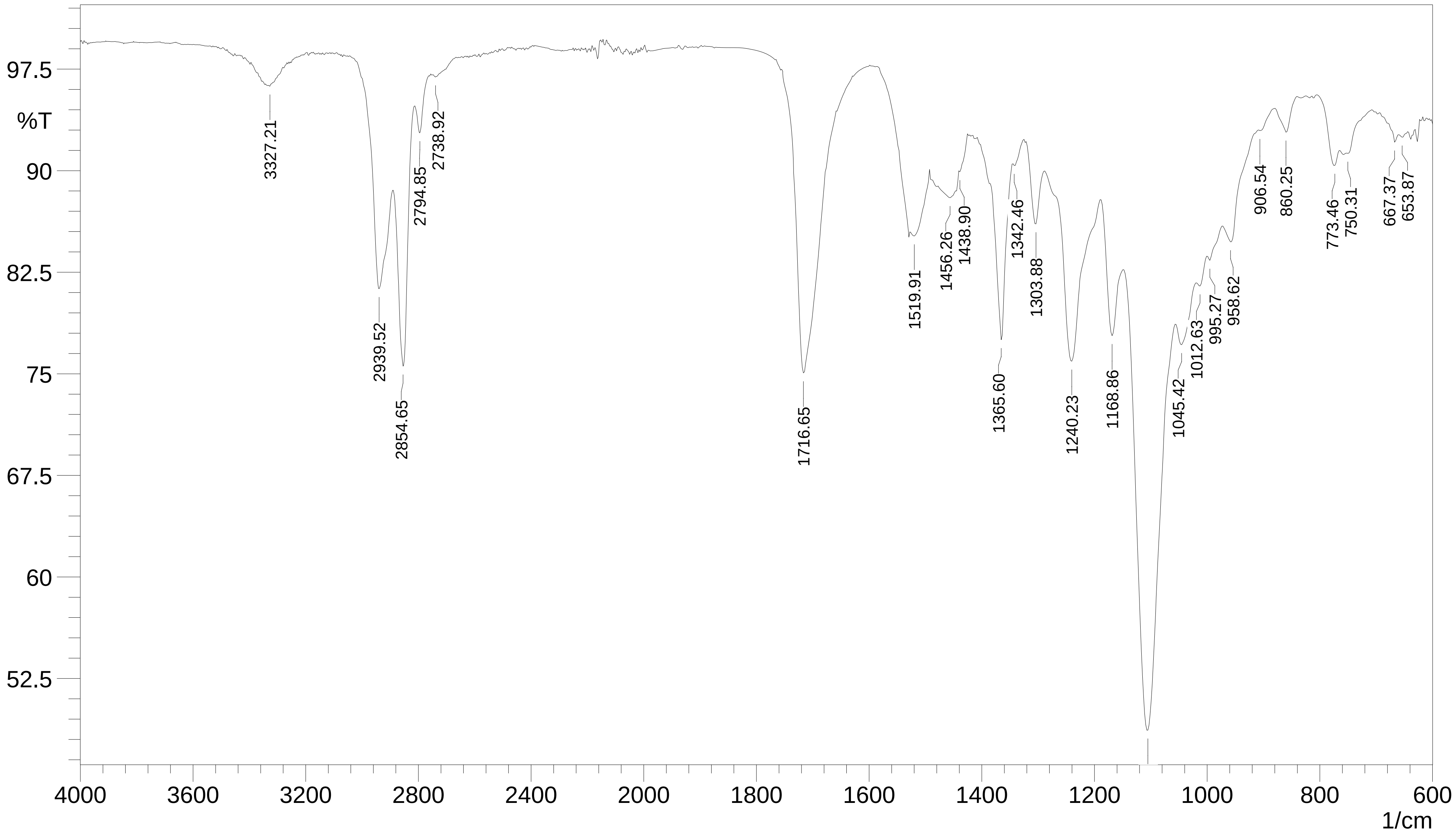

No. of Scans; 20
Resolution; 4 [1/cm]
Apodization; Happ-Genzel

PUI-A1

Figure S18: ATR-FT-IR

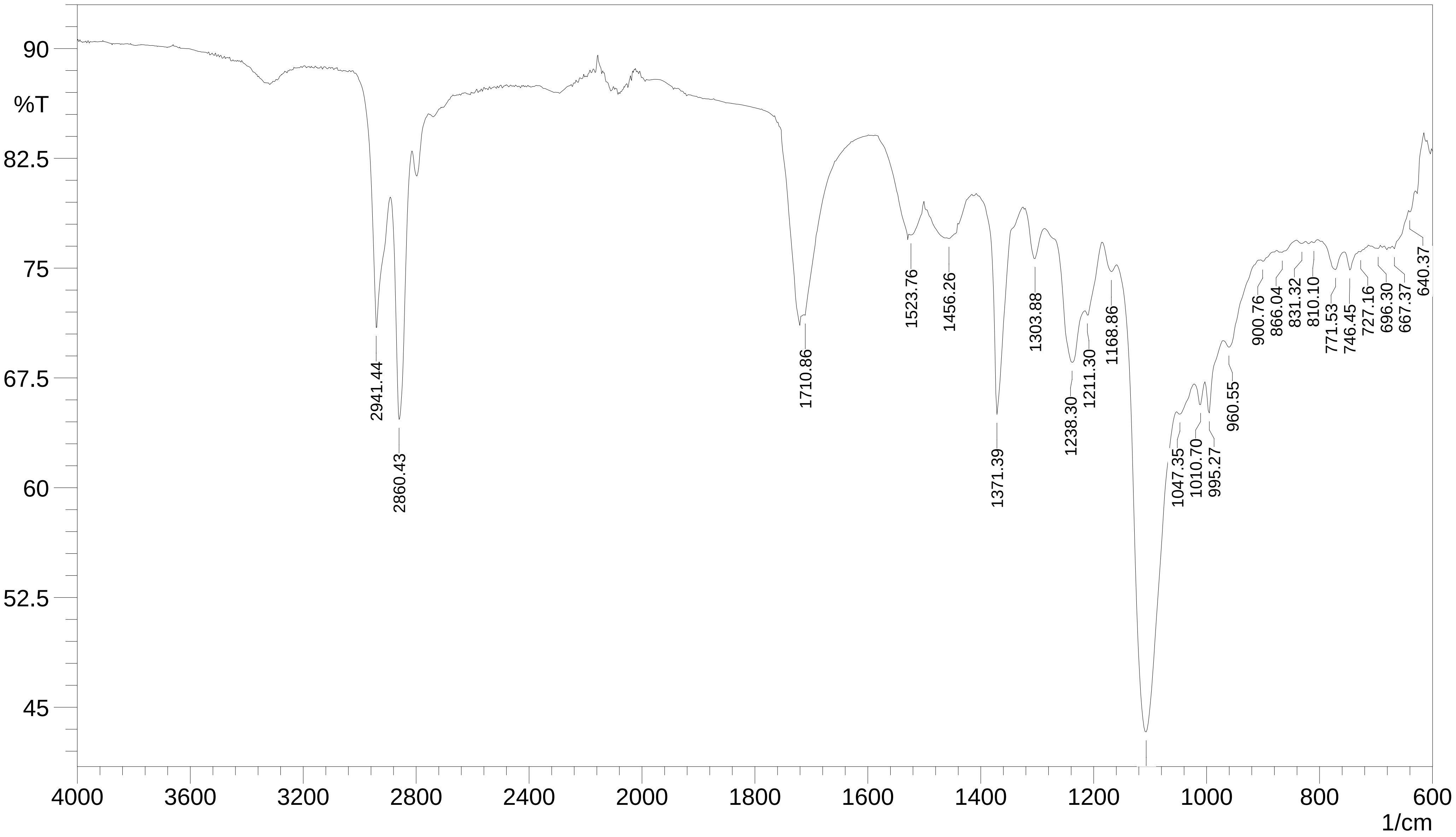

No. of Scans; 20
Resolution; 4 [1/cm]
Apodization; Happ-Genzel

PUI-A2

Figure S19: ATR-FT-IR

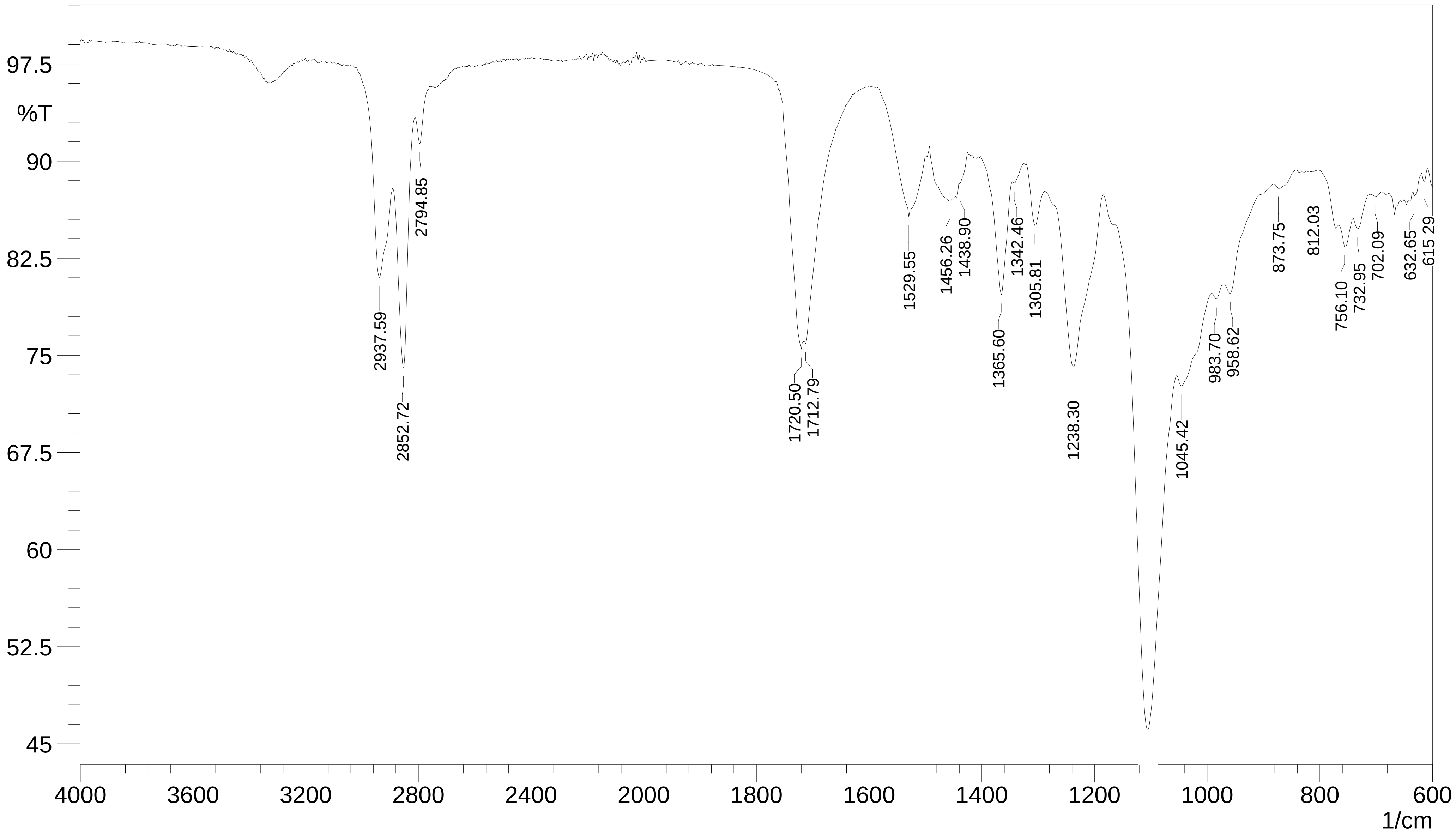

No. of Scans; 20
Resolution; 4 [1/cm]
Apodization; Happ-Genzel

PUI-S2

Figure S20: ATR-FT-IR

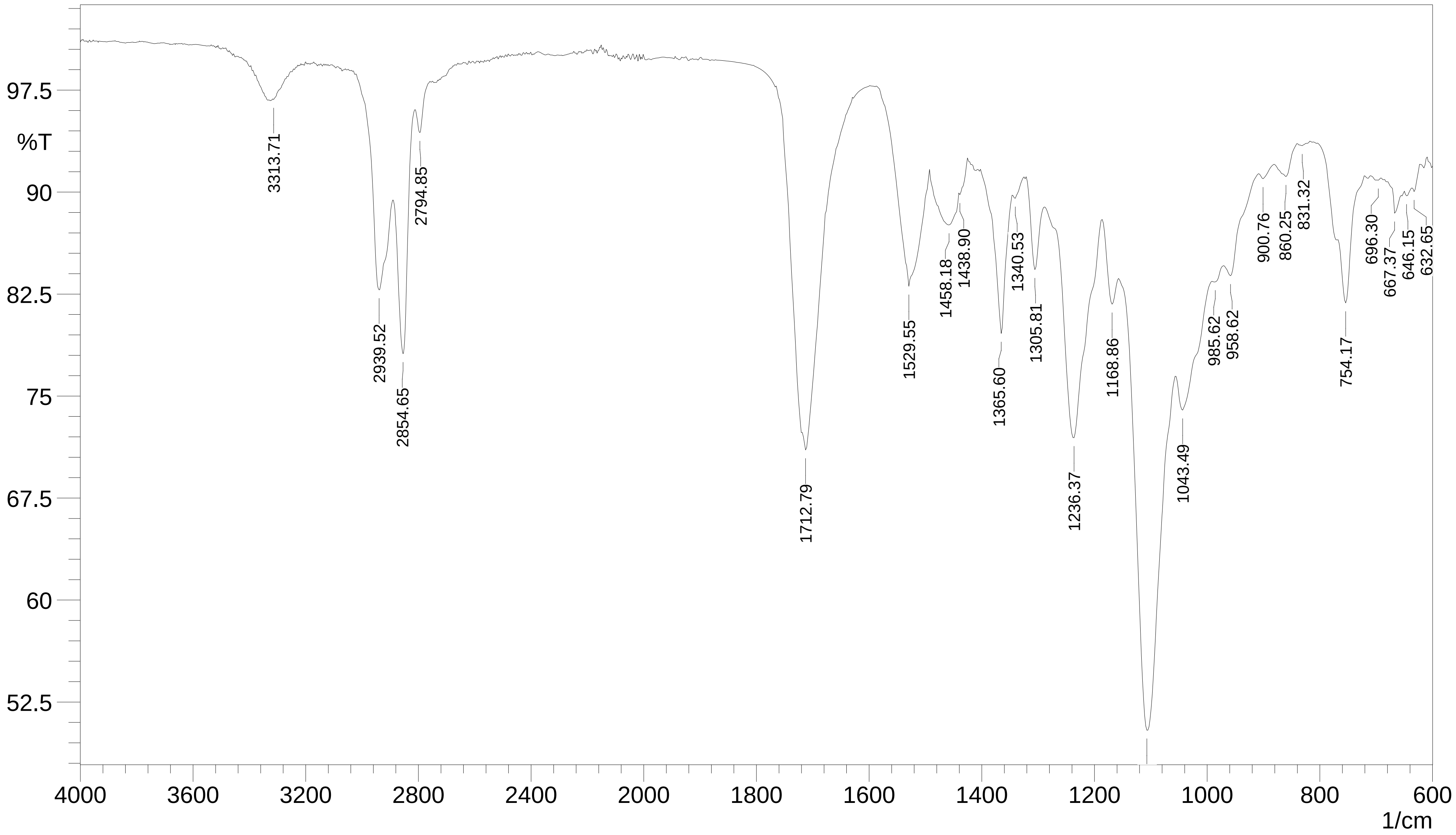

No. of Scans; 20

Resolution; 4 [1/cm]

Apodization; Happ-Genzel

Figure S21: DSC

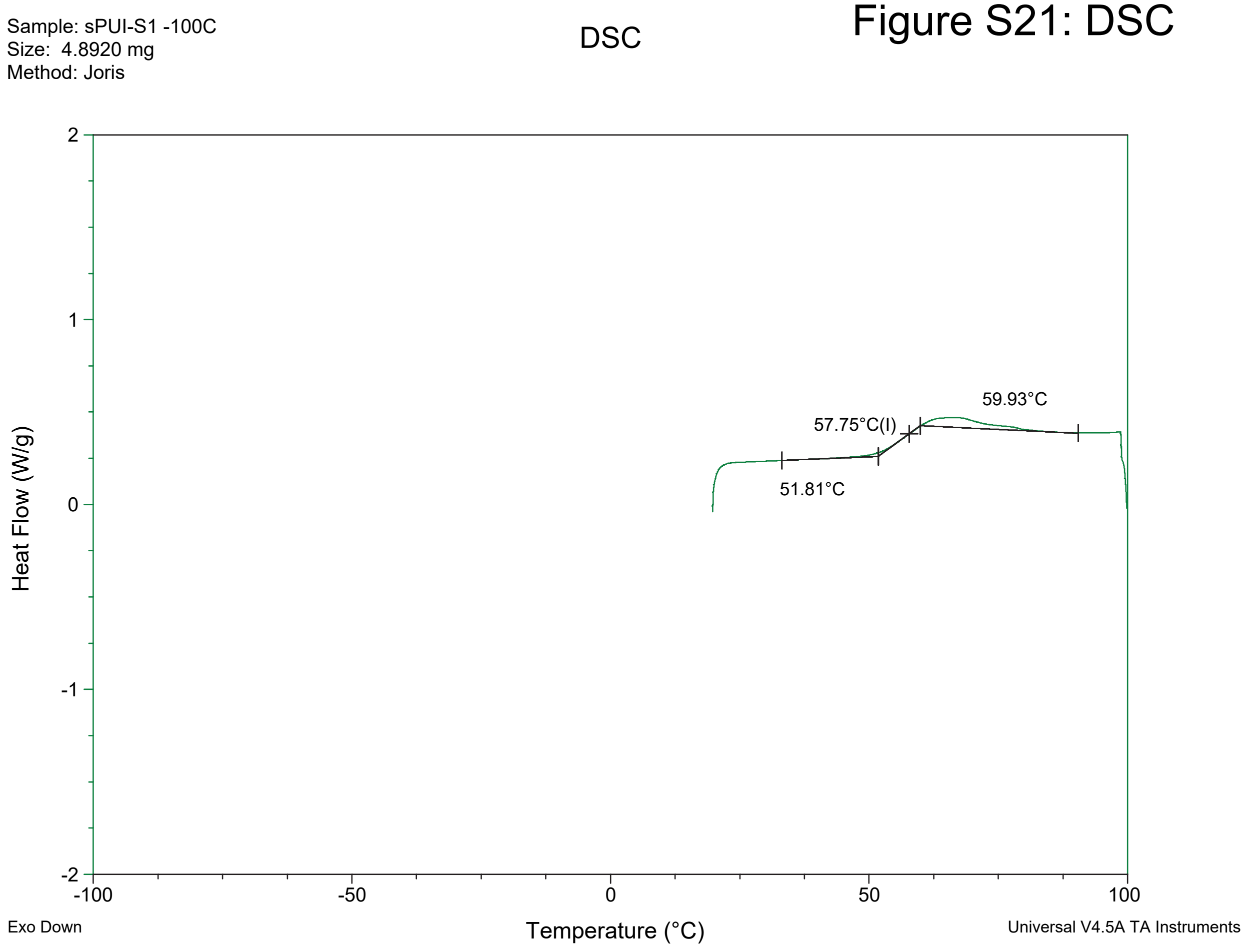

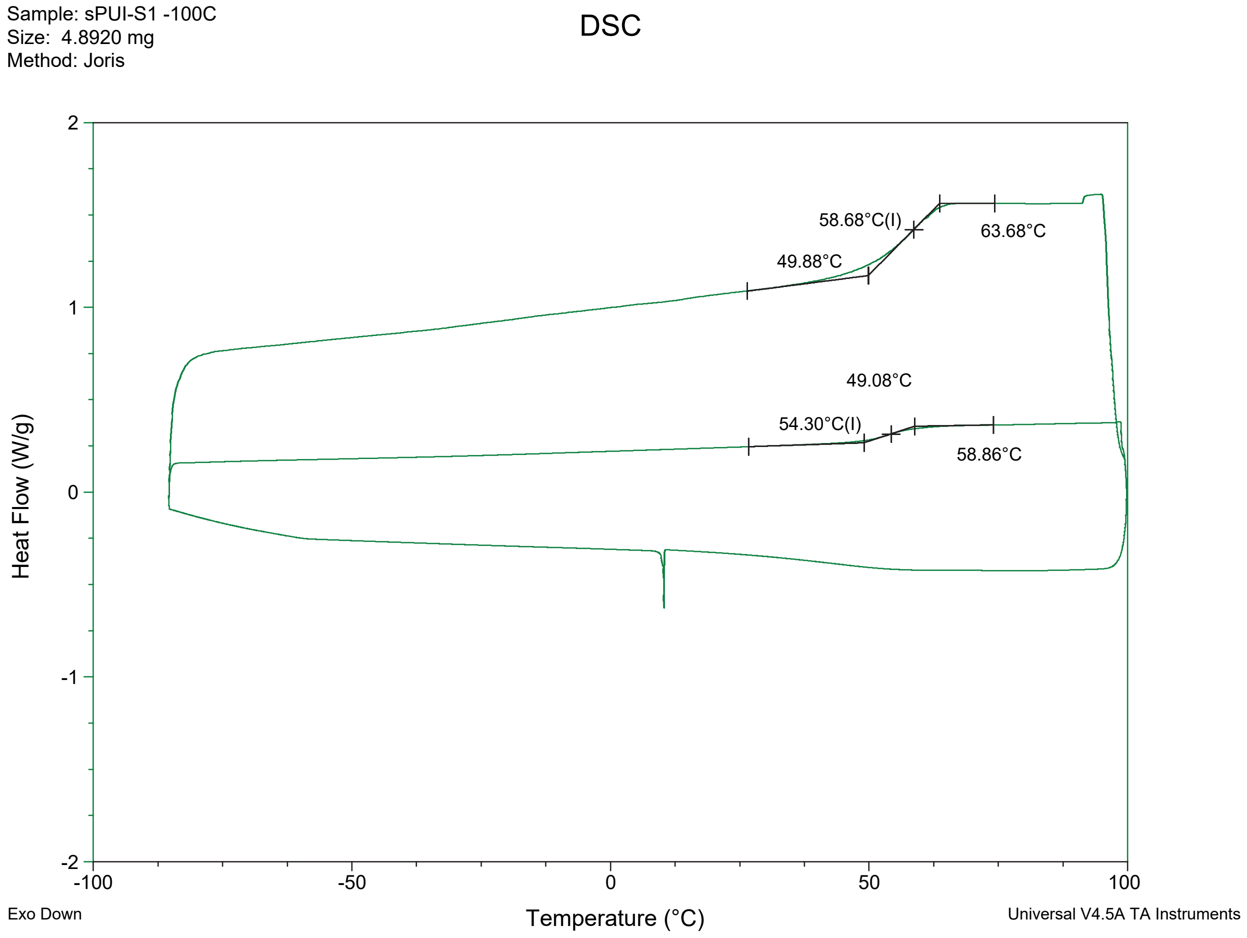

Figure S22: DSC

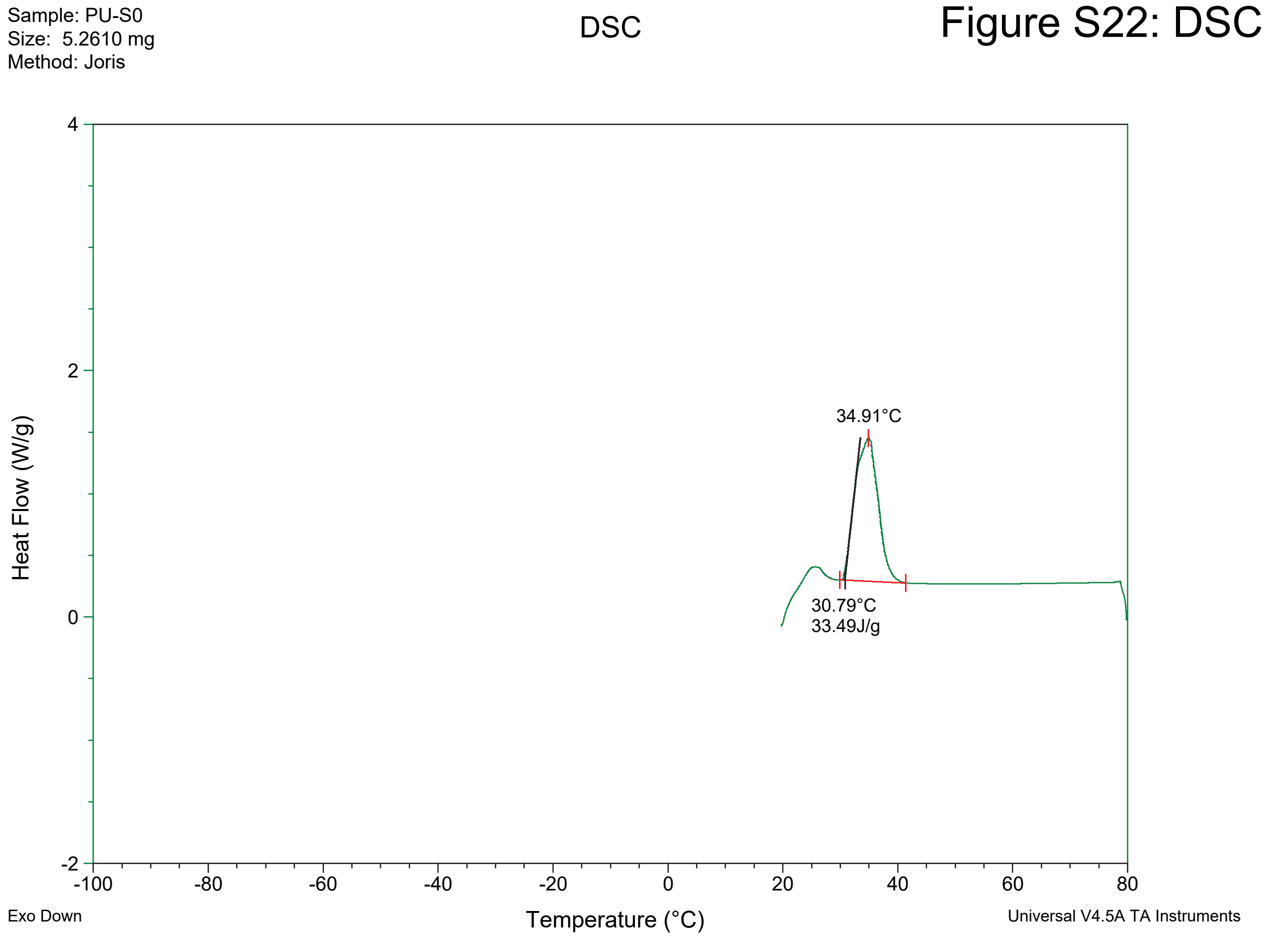

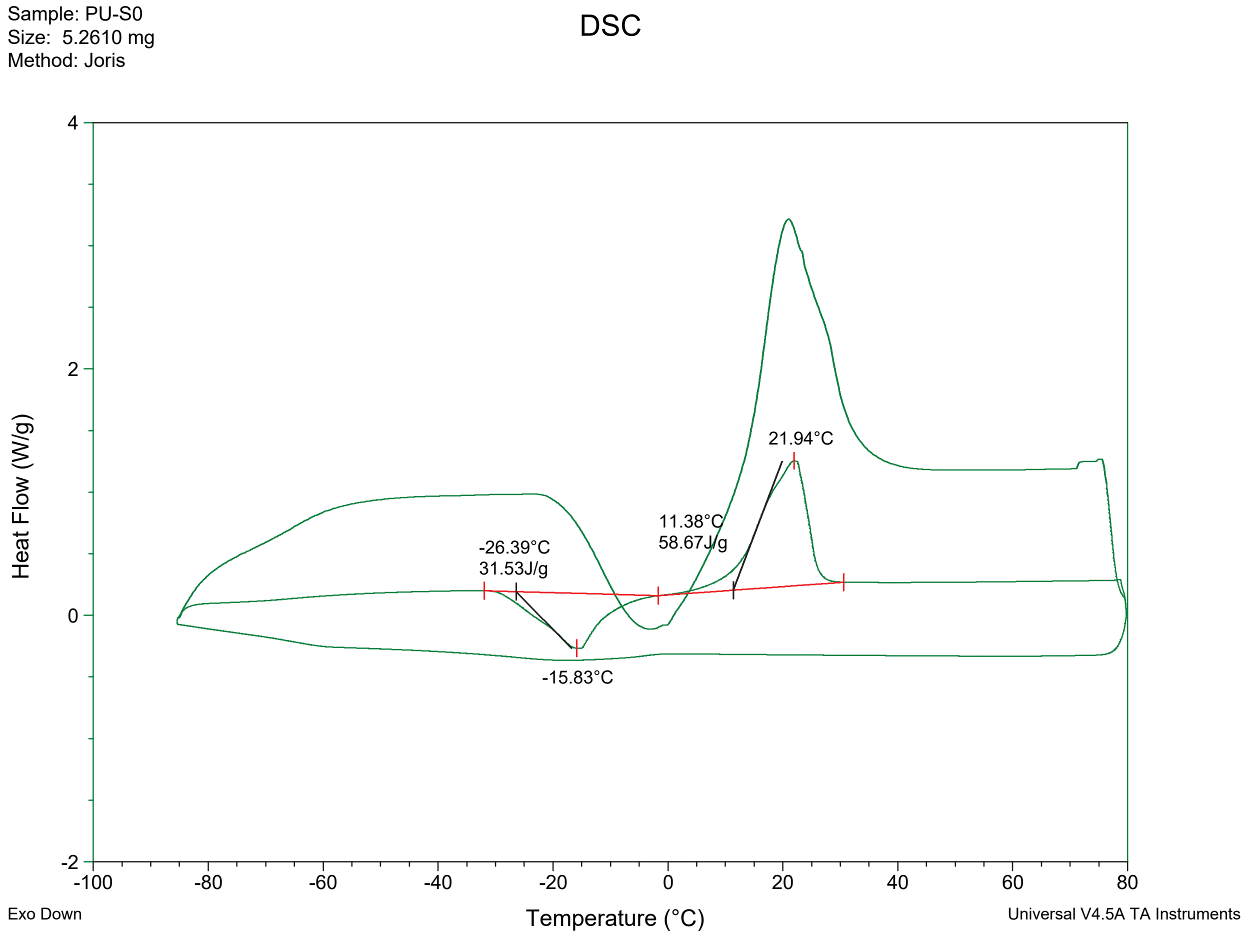

Sample: PUI-A1
Size: 5.3330 mg
Method: Joris

DSC

Figure S23: DSC

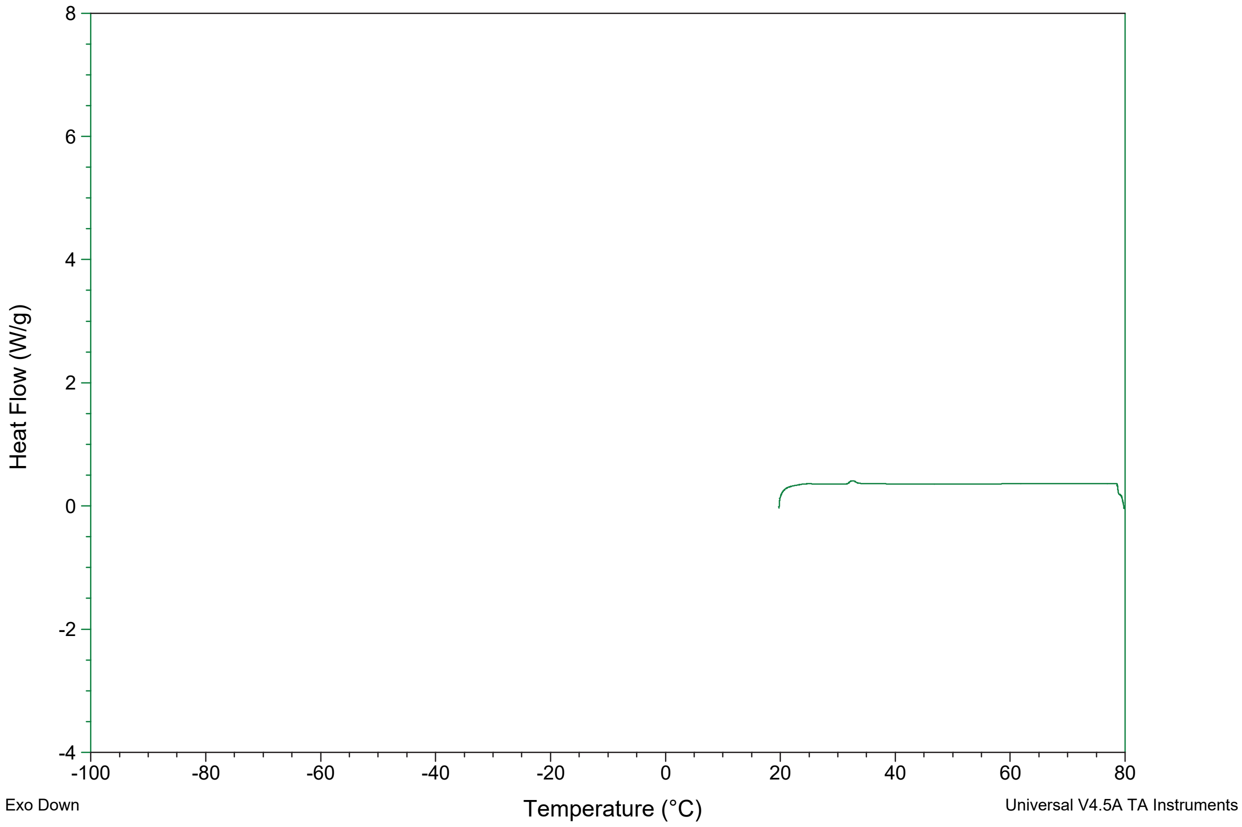

Sample: PUI-A1
Size: 5.3330 mg
Method: Joris

DSC

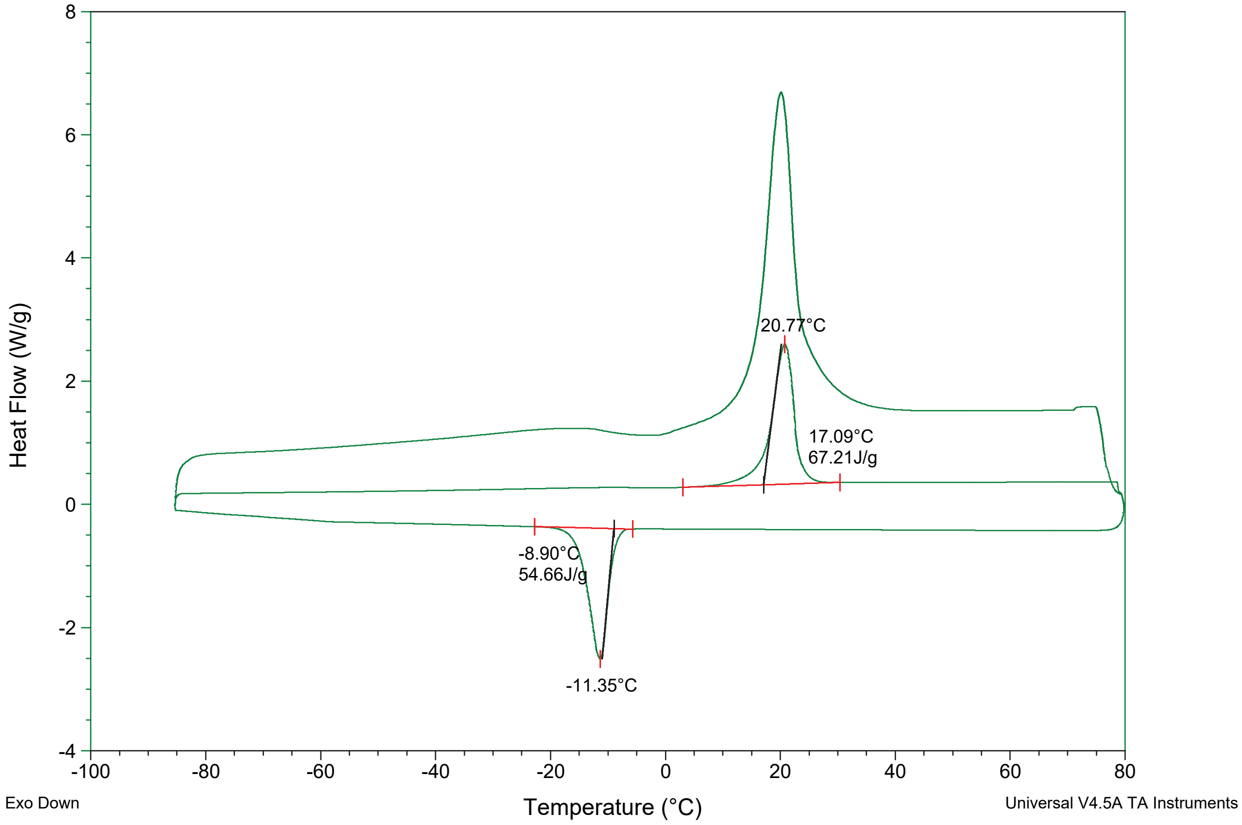

Figure S24: DSC

Sample: PUI-A2
Size: 5.5400 mg
Method: Joris

DSC

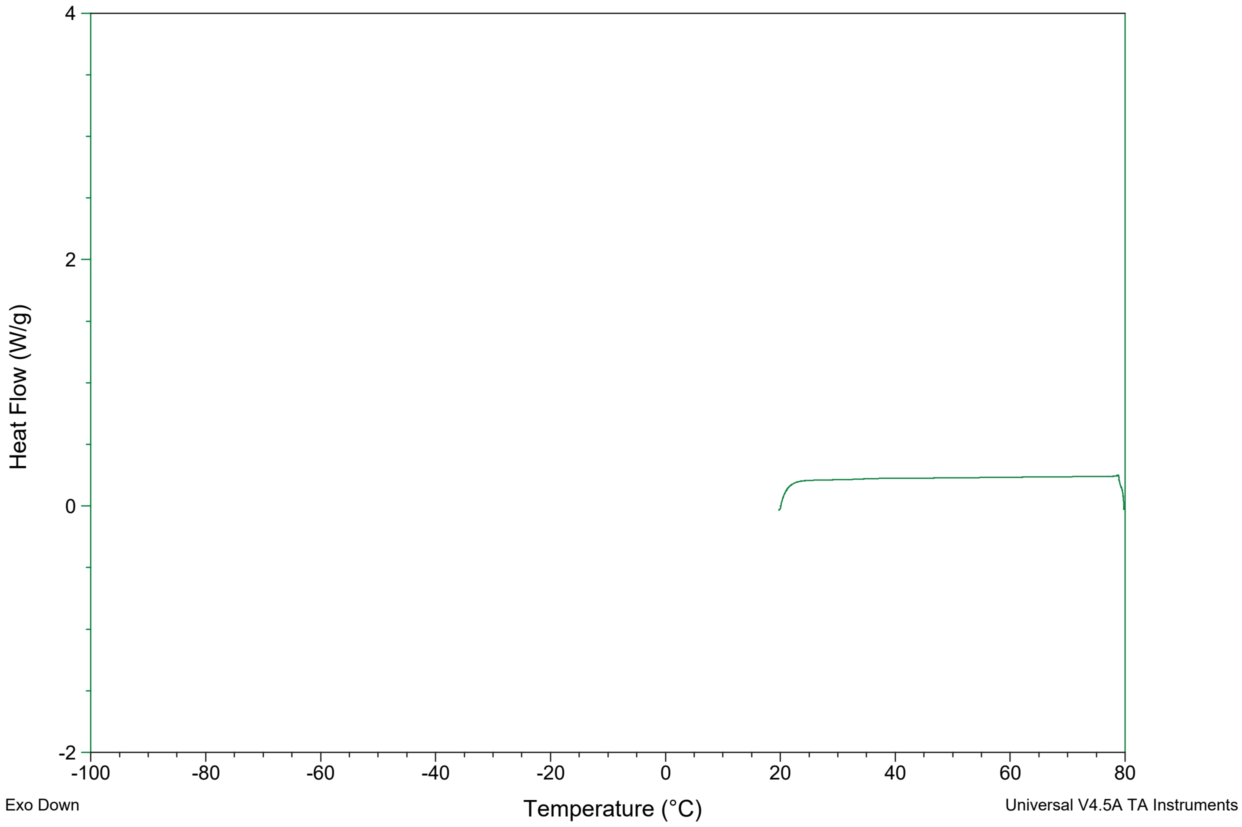

Sample: PUI-A2
Size: 5.5400 mg
Method: Joris

DSC

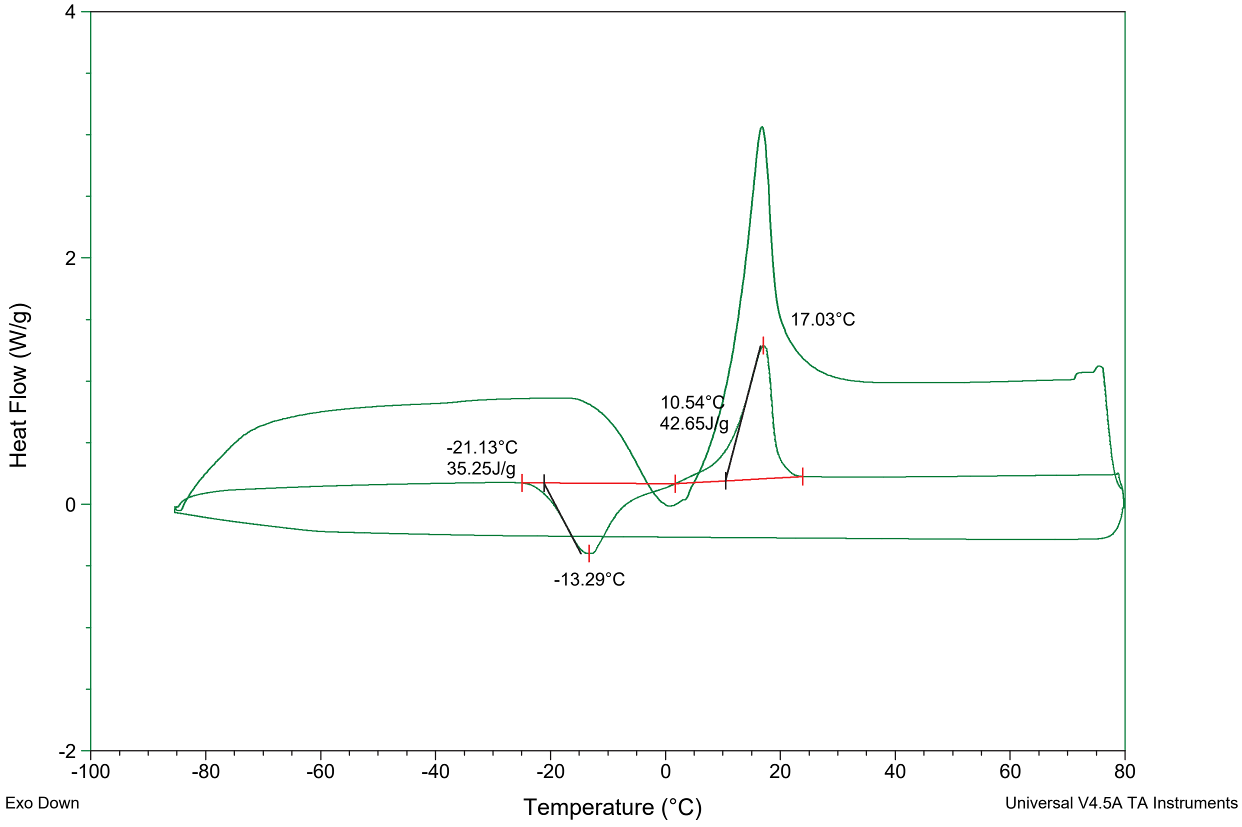

Figure S25: DSC

Sample: PUI-S2
Size: 6.0070 mg
Method: Joris

DSC

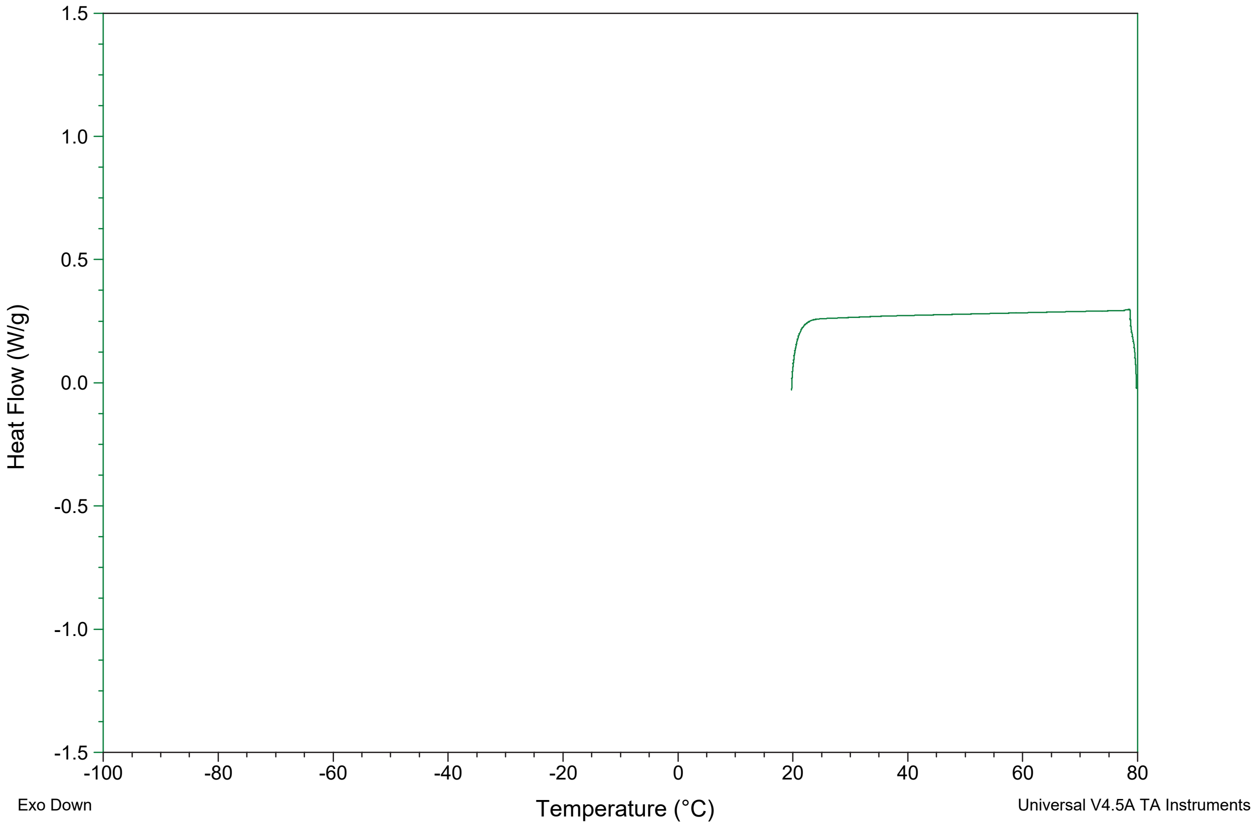

Sample: PUI-S2
Size: 6.0070 mg
Method: Joris

DSC

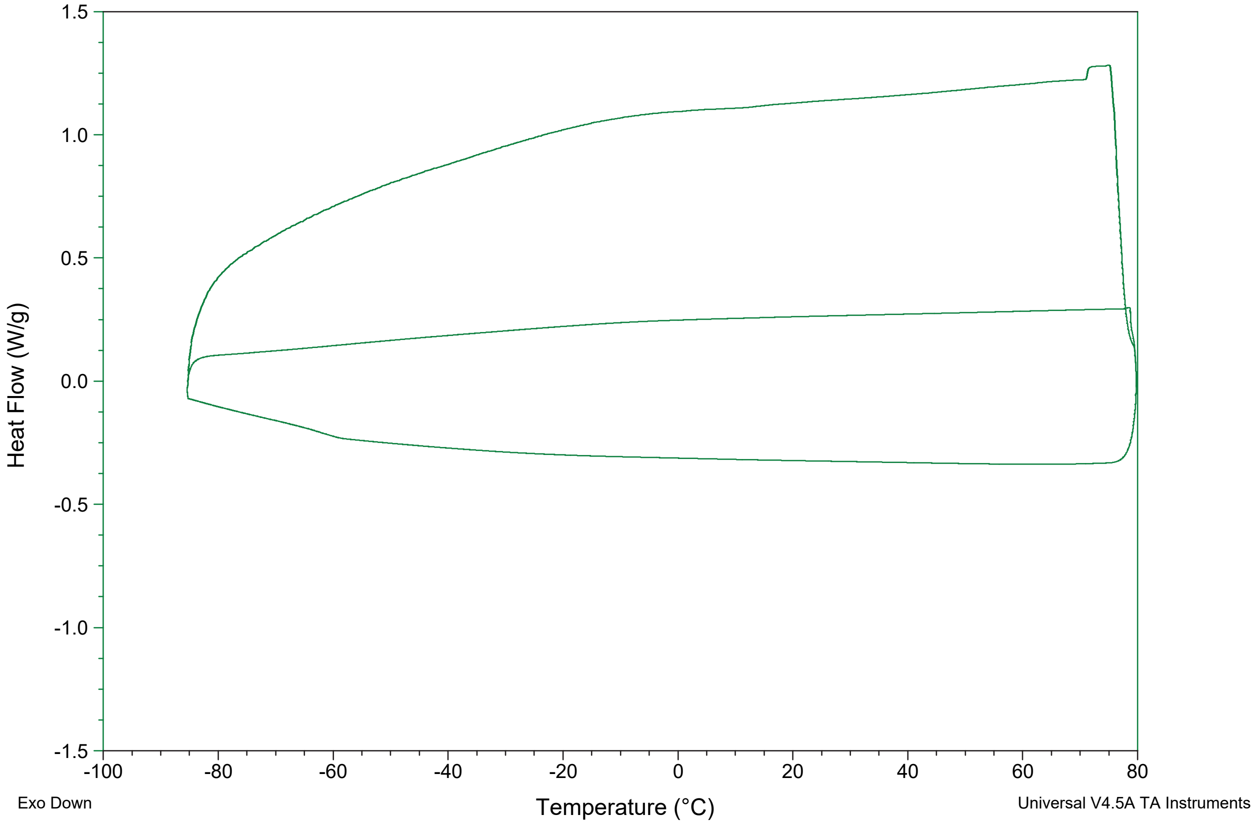

Supplement: PY-012-D1PY00079A-s001 [file PY-012-D1PY00079A-s001.pdf]
